# Tartrate-resistant acid phosphatase 5 promotes pulmonary fibrosis by modulating β-catenin signaling

**DOI:** 10.1038/s41467-021-27684-9

**Published:** 2022-01-10

**Authors:** Yinan Hu, Qi Wang, Jun Yu, Qing Zhou, Yanhan Deng, Juan Liu, Lei Zhang, Yongjian Xu, Weining Xiong, Yi Wang

**Affiliations:** 1 Department of Pulmonary and Critical Care Medicine, NHC Key Laboratory of Respiratory Diseases, Key Site of National Clinical Research Center for Respiratory Disease, Wuhan Clinical Medical Research Center for Chronic Airway Diseases, Tongji Hospital, Tongji Medical College, Huazhong University of Science and Technology, 1095 Jiefang Ave, Wuhan, 430030 China; 2 Department of Pulmonary and Critical Care Medicine, Center of Respiratory Medicine, National Center for Respiratory Medicine, Institute of Respiratory Medicine, Chinese Academy of Medical Sciences, National Clinical Research Center for Respiratory Diseases, China-Japan Friendship Hospital, 100029 Beijing, China; 3 Department of Thoracic Surgery, Tongji Hospital, Tongji Medical College, Huazhong University of Science and Technology, 1095 Jiefang Ave, Wuhan, 430030 China; 4 The Center for Biomedical Research, Tongji Hospital, Tongji Medical College, Huazhong University of Science and Technology, 1095 Jiefang Ave, Wuhan, 430030 China; 5 Department of Pulmonary and Critical Care Medicine, Shanghai Key Laboratory of Tissue Engineering, Shanghai Ninth People’s Hospital, Shanghai Jiaotong University School of Medicine, 639 Zhizaoju Lu, Shanghai, 200011 China

**Keywords:** Cell signalling, Mechanisms of disease, Respiratory tract diseases

## Abstract

Idiopathic pulmonary fibrosis (IPF) is a fatal interstitial lung disease with limited therapeutic options. Tartrate-resistant acid phosphatase 5 (ACP5) performs a variety of functions. However, its role in IPF remains unclear. Here, we demonstrate that the levels of ACP5 are increased in IPF patient samples and mice with bleomycin (BLM)-induced pulmonary fibrosis. In particular, higher levels of ACP5 are present in the sera of IPF patients with a diffusing capacity of the lungs for carbonmonoxide (DLCO) less than 40% of the predicted value. Additionally, *Acp5* deficiency protects mice from BLM-induced lung injury and fibrosis coupled with a significant reduction of fibroblast differentiation and proliferation. Mechanistic studies reveal that Acp5 is upregulated by transforming growth factor-β1 (TGF-β1) in a TGF-β receptor 1 (TGFβR1)/Smad family member 3 (Smad3)-dependent manner, after which Acp5 dephosphorylates p-β-catenin at serine 33 and threonine 41, inhibiting the degradation of β-catenin and subsequently enhancing β-catenin signaling in the nucleus, which promotes the differentiation, proliferation and migration of fibroblast. More importantly, the treatment of mice with *Acp5* siRNA-loaded liposomes or Acp5 inhibitor reverses established lung fibrosis. In conclusions, Acp5 is involved in the initiation and progression of pulmonary fibrosis and strategies aimed at silencing or suppressing *Acp5* could be considered as potential therapeutic approaches against pulmonary fibrosis.

## Abstract (web summary)

Idiopathic pulmonary fibrosis is a fatal lung disease with limited treatment options. Here the authors show that tartrate-resistant acid phosphatase 5 (Acp5) promotes lung fibrosis by enhancing beta-catenin signaling and that inhibition of Acp5 can reverse stablished pulmonary fibrosis.

## Introduction

Idiopathic pulmonary fibrosis (IPF) is a fatal interstitial lung disease characterized by the deposition of excessive extracellular matrix (ECM), destruction of the lung parenchyma and a pattern of usual interstitial pneumonia (UIP) by radiology and histopathology^1^. Although the development of new antifibrotic agents (pirfenidone and nintedanib) has improved patients wellbeing, the incidence and mortality rate of IPF have barely improved, resulting in a median survival time following diagnosis only 3–5 years^2,3^. Therefore, exploration of the pathogenesis of IPF and the discovery of new therapeutic methods for IPF patients are urgently needed.

According to the current paradigm, the main pathological features of IPF include epithelial injury^4^, the recruitment of inflammatory cells^5,6^, the aberrant differentiation and proliferation of fibroblasts^7^ and the persistence of apoptosis-resistant myofibroblasts^8^ in fibrotic lesions. Resident lung fibroblasts-derived myofibroblasts are the major contributors to the processes of ECM deposition and tissue distortion in IPF^9,10^. Under stimulation with fibrotic factors, such as transforming growth factor-β (TGF-β)^11^, platelet-derived growth factor (PDGF)^12^ and connective tissue growth factor (CTGF)^13^, resident fibroblasts in the lung lesion transform into myofibroblasts, which are characterized by a spindle or stellate morphology with α-smooth muscle actin (α-SMA) stress fibers coupled with a hypersecretion phenotype due to which they produce copious amounts of fibrillary ECM proteins, such as Collagen and Fibronectin.

Tartrate-Resistant Acid Phosphatase 5 (TRAP/ACP5), also named purple acid phosphatase, which locates on chromosome 19 band p13.2–p13.3 locus in human being, contains a binuclear iron center (NCBI GENE, https://www.ncbi.nlm.nih.gov/gene/54)^14^. Although encoded by a single gene, ACP5 exists as two isoforms, ACP5a and ACP5b^15^. It is well noted that ACP5a exhibits properties of a proenzyme with low phosphatase activity, while ACP5b, derived from ACP5a by proteolytic cleavage in the intervening loop domain with serine proteases or cathepsin, displays high phosphatase activity^16^. There was feeble evidence that Acp5 was highly expressed in the early stages of liver fibrosis progression^17^. Previously, ACP5 was demonstrated to interact intracellularly with the TGFβ receptor interacting protein-1 (TRIP-1), thereby activating TGFβ receptor type 2 (TGFβR2) & Smad2/3 signaling, leading to the metastasis of breast cancer cells^18^. Similarly, our study identified that ACP5 promoted A549 and NCI-H1975 cells undergoing epithelial-mesenchymal transition (EMT) via P53/Smad3 signaling^19^. Given that TGF-β/Smads signaling plays a critical role in the process of fibroblast to myofibroblast transition, we hypothesize that ACP5 is involved in the pathogenesis of IPF.

To assess the feasibility of this hypothesis, we first detected the expression of ACP5 in IPF patients and mice with bleomycin (BLM)-induced pulmonary fibrosis. Notably, the levels of ACP5 in the sera of IPF patients were correlated with disease severity. Furthermore, the loss of *Acp5* (NCBI GENE, https://www.ncbi.nlm.nih.gov/gene/11433) significantly protected mice from BLM-induced lung injury and fibrosis, accompanied by a marked reduction in myofibroblast accumulation. Mechanistically, Acp5 selectively bound and dephosphorylated β-catenin at serine (Ser) 33 and threonine (Thr) 41 in the cytoplasm, and then reduced the degradation of β-catenin, which enhanced the levels of β-catenin in the nucleus to promote fibroblast differentiation, proliferation and migration. Therefore, intratracheal administration of liposomes carrying *Acp5* siRNA or intraperitoneal injection of Acp5 inhibitor, AubipyOMe, significantly reversed the established pulmonary fibrosis induced by BLM. Collectively, our data support the notion that ACP5 is involved in the progression of pulmonary fibrosis; therefore, strategies aimed at silencing or suppressing Acp5 could be viable therapies against pulmonary fibrosis in clinical settings.

## Result

### IPF is characterized by altered ACP5 expression

**Fig. 1:**
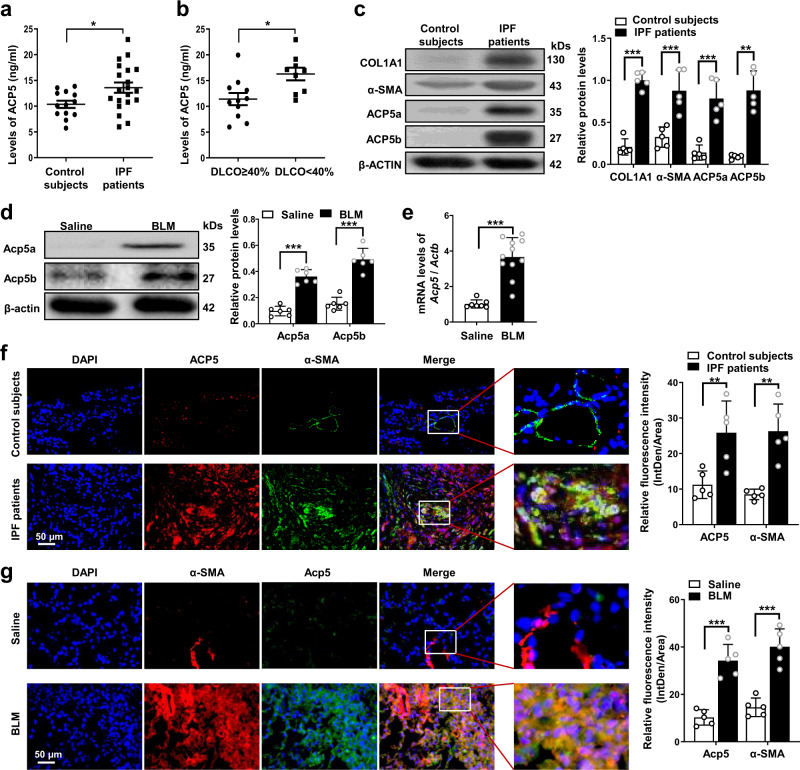
Analysis of ACP5 levels in Idiopathic pulmonary fibrosis (IPF) patients and mice with bleomycin (BLM) induction. **a** ELISA analysis of ACP5 levels in the serum of patients with IPF (*n* = 20) and control subjects (*n* = 13, *p* = 0.0134). **b** Analysis of the correlation between ACP5 levels with diffusion capacity carbonmonoxide lung (DLCO)% predicted in IPF patients (*n* = 20, *p* = 0.0113). **c** Western blot analysis of ACP5a (*p* = 0.002) and ACP5b (*p* = 0.0015), COL1A1 (*p* < 0.001) and α-SMA (*p* = 0.001) expression in the lungs of control subjects (*n* = 5) and IPF patients (*n* = 5). **d** Western blot analysis of Acp5a (*p* < 0.001) and Acp5b (*p* < 0.001) expression in the lung homogenate of Saline (*n* = 6) and BLM-induced (*n* = 6) mouse model. **e** RT-PCR analysis of *Acp5* expression in the lung homogenate from Saline (n = 6) and BLM-induced (n = 11) mice (*p* < 0.001). **f** Representative results for coimmunostaining of ACP5 (*p* = 0.0095) and α-SMA (a myofibroblast marker, *p* = 0.0059) in the lung sections from patients with IPF (*n* = 5) and control subjects (*n* = 5). **g** Results for coimmunostaining of Acp5 (*p* < 0.001) and α-SMA (*p* < 0.001) in the lung sections from Saline (*n* = 5) and BLM-induced (*n* = 5) mice. The nuclei were stained blue by DAPI, and the images were taken under original magnification ×400. The data are represented as the mean ± SEM. Two-sided unpaired Student’s *t* test with Welch’s correction (**a, c, d, f, g**) and two-sided Student’s *t* test (**b, c, e, f**) were applied. *p < 0.05; **p < 0.01; ***p < 0.001. Source data are provided as a Source Data file.

We first sought to examine the levels of ACP5 in the sera of IPF patients and control subjects. Interestingly, ACP5 levels in the serum derived from control subjects were low, and higher levels of ACP5 were detected in the serum samples from IPF patients (Fig. 1a). Then, we assessed the correlation between the levels of ACP5 and disease severity, which were estimated by the lung’s gas diffusing capacity^20^ (as measured by the diffusing capacity of the lungs for carbonmonoxide, DLCO). Notably, the levels of ACP5 were higher in severe IPF patients whose DLCO were less than 40% of the predicted value than in patients with less severe IPF (Fig. 1b). To further address the role of ACP5 in pulmonary fibrosis, we then detected the expression of ACP5 in the lungs of IPF patients and mice with the onset of BLM-induced pulmonary fibrosis. Interestingly, the lung homogenates of IPF patients exhibited 6.4-fold and 10.8-fold higher ACP5a and ACP5b expression than those of control subjects, respectively and markedly higher expression levels of COL1A1 and α-SMA, markers of fibrosis, were also noted in IPF patients (Fig. 1c). Similarly, significantly higher Acp5a and Acp5b expression were detected in the lungs of mice following BLM injection than in those of saline-treated mice (Fig. 1d). Consistent results were also obtained by RT-PCR analysis of *Acp5* expression (Fig. 1e). Next, we sought to examine cells showing altered ACP5 expression in the lung sections from IPF patients and control subjects. Coimmunostaining showed that ACP5 was almost undetectable in the lung sections from control subjects, while IPF patient-derived lung sections were characterized by a large amount of lung myofibroblast aggregation, manifesting as high levels of ACP5 revealed by costaining for ACP5 with α-SMA (Fig. 1f). We further detected lung sections from mice after BLM injection and found that the progression of pulmonary fibrosis was highly correlated with the severity of myofibroblast accumulation and Acp5 overexpression in the lungs (Fig. 1g).

### Acp5 is upregulated in a canonical TGF-β signaling-dependent manner

**Fig. 2:**
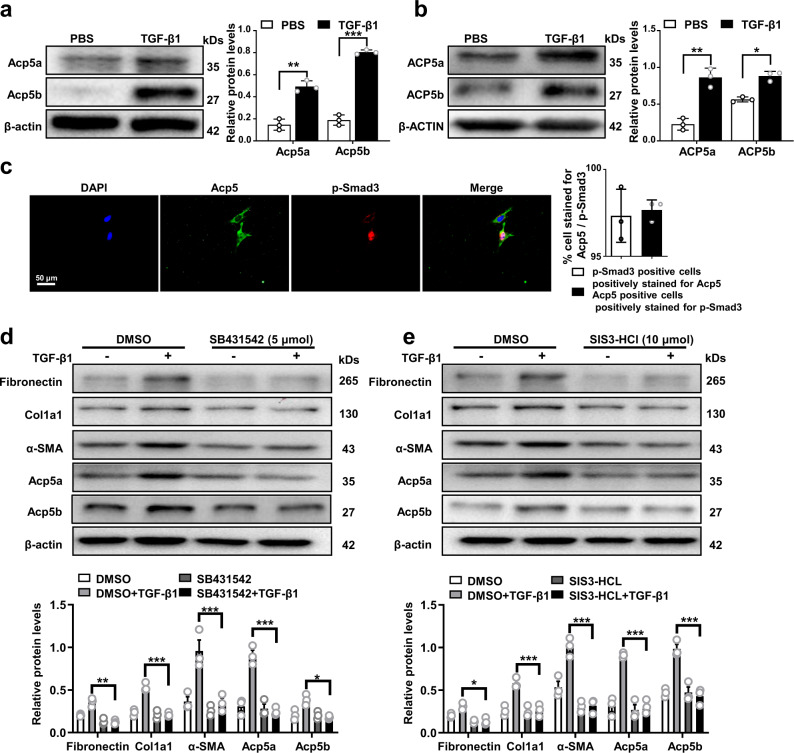
Acp5 is upregulated in fibroblasts in a transforming growth factor β receptor 1 (TGFβR1) / Smad family member 3 (Smad3)-dependent manner. **a** Western blot analysis of Acp5a (*p* = 0.0029) and Acp5b (*p* = 0.0003) expression in primary mice lung fibroblasts (PMLFs) following TGF-β1 induction. **b** Western blot analysis of ACP5a (*p* = 0.0040) and ACP5b (*p* = 0.0455) expression in primary human lung fibroblasts (PHLFs) following TGF-β1 induction. **c** Representative results for coimmunostaining of Acp5 and p-Smad3 in PMLFs. The nuclei were stained blue by DAPI, and the images were taken under original magnification ×400. **d–e** Western blot analysis of Fibronectin (**d**: *p* = 0.0096, **e**: *p* = 0.0295), Col1a1 (**d**: *p* = 0.0003, **e**: *p* < 0.0001), α-SMA (**d**: *p* < 0.0001, **e**: *p* < 0.0001), Acp5a (**d**: *p* < 0.0001, **e**: *p* < 0.0001) and Acp5b (**d**: *p* = 0.02, **e**: *p* < 0.0001) expression in PMLFs following SB431542 (**d**) and SIS3-HCL (**e**) treatment. The data are represented as the mean ± SEM of three independent experiments and two-side Student’s *t*-test was administered to analyze the statistical significance of differences between two groups. *p < 0.05; **p < 0.01; ***p < 0.001. Source data are provided as a Source Data file.

To further confirm the above results, we cultured primary lung fibroblasts from WT mice and control individuals and then stimulated them with TGF-β1. Indeed, low levels of ACP5a and ACP5b were observed in fibroblasts before stimulation. Upon TGF-β1 stimulation, a substantial increase in ACP5a and ACP5b levels in primary mouse pulmonary fibroblasts (PMLFs) (Fig. 2a) and primary human pulmonary fibroblasts (PHLFs) (Fig. 2b) was noted. Given that TGF-β/Smad3 signaling plays a pivotal role in fibroblast differentiation induced by TGF-β1^21^, we conducted coimmunostaining for Acp5 and p-Smad3 in PMLFs. Interestingly, Acp5 colocalized with p-Smad3 in cultured fibroblasts (Fig. 2c). These findings suggested to us that Acp5 may be a downstream target of canonical TGF-β signaling. To assess this hypothesis, we treated PMLFs with TGF-β1 and SB431542, an inhibitor of TGF-β receptor 1 (TGFβR1). As expected, the expression of Fibronectin, Col1a1 and α-SMA was significantly enhanced following TGF-β1 treatment (Fig. 2d). However, inhibition of TGFβR1 activation by SB431542 absolutely abolished the upregulation of these fibrotic proteins. Surprisingly, the overexpression of Acp5a and Acp5b induced by TGF-β1 was reversed after SB431542 treatment (Fig. 2d). Interestingly, similar results were observed when PMLFs were stimulated with TGF-β1 and SIS3-HCl (an inhibitor of Smad3 phosphorylation) (Fig. 2e). Collectively, these data support the notion that Acp5 expression in fibroblasts is controlled by canonical TGF-β signaling, of which TGFβR1/Smad3 are essential mediators.

### ACP5 facilitates fibroblast differentiation, proliferation and migration

**Fig. 3:**
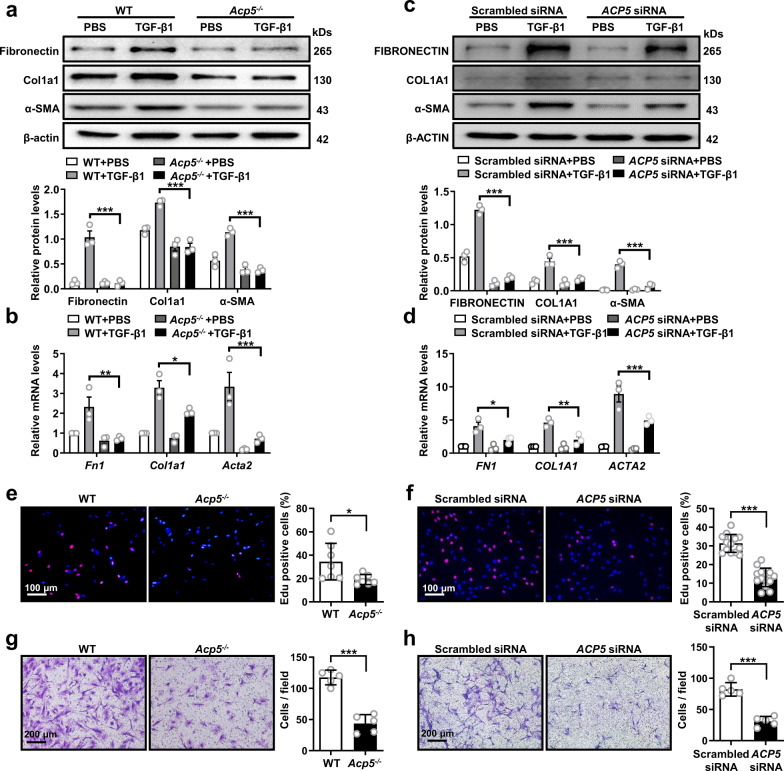
The impact of *ACP5* on the differentiation, proliferation and migration of fibroblasts. **a**, **b** Western blot (**a**) and RT-PCR (**b**) analysis of Fibronectin (**a**: *p* < 0.0001, **b**: *p* = 0.0038), Col1a1 (**a**: *p* < 0.0001, **b**: *p* = 0.0284) and α-SMA (**a**: *p* < 0.0001, **b**: *p* < 0.0001) expression in PMLFs from WT or *Acp5*^*−/−*^ mice following TGF-β1 treatment**. c**, **d** Western blot (**c**) and RT-PCR (**d**) analysis of FIBRONECTIN (**c**: *p* < 0.0001, **d**: *p* = 0.0213), COL1A1 (**c**: *p* < 0.0001, **d**: *p* = 0.0041) and α-SMA (**c**: *p* < 0.0001, **d**: *p* < 0.0001) expression in *ACP5* siRNA or Scrambled siRNA treated PHLFs following TGF-β1 induction. **e, f** Representative results for EdU staining in WT or *Acp5*^*−/−*^ PMLFs (**e**, *p* = 0.0421) and *ACP5* siRNA or Scrambled siRNA treated PHLFs (**f**, *p* < 0.0001) **g, h** Representative results for Transwell assay in WT or *Acp5*^*−/−*^ mice derived PMLFs (**g**, *p* < 0.0001) and *ACP5* siRNA or Scrambled siRNA treated PHLFs (**h**, *p* < 0.0001). The data are represented as the mean ± SEM of three independent experiments. Two-sided Student’s *t* test (**a**–**d, f**–**h**) and two-sided unpaired Student’s *t*-test with Welch’s correction (e) were applied. *p < 0.05; **p < 0.01; ***p < 0.001. Source data are provided as a Source Data file.

To assess the functional role of ACP5 in lung fibroblasts, PMLFs were generated from WT and *Acp5*^*−/−*^ mice and then subjected to TGF-β1 stimulation. Compared with fibroblast differentiation in WT PMLFs, loss of *Acp5* significantly attenuated the differentiation of fibroblast into myofibroblast, as evidenced by the decreased expression of myofibroblast markers (Fibronectin, Col1a1 and α-SMA) following TGF-β1 treatment (Fig. 3a). Consistently, RT-PCR analysis of the expression of these genes demonstrated similar results (Fig. 3b). To further confirm the above data, we then performed ACP5 gain- and loss-of-function assays in PHLFs following TGF-β1 treatment. As expected, the expression of *ACP5* was efficiently silenced or enhanced following *ACP5* siRNA or *ACP5-*plasmid transfection (Supplementary Fig. 1a, b). Notably, both Western blot and RT-PCR analyses demonstrated that fibroblast differentiation to myofibroblast was abrogated in *ACP5* siRNA-transfected PHLFs after TGF-β1 induction (Fig. 3c, d), while a significant increase in myofibroblast markers was observed in *ACP5*-overexpressing PHLFs (Supplementary Fig. 1c). Additionally, we estimated the impact of ACP5 on the proliferation of PMLFs and PHLFs by EdU staining and CFSE staining, respectively. Fewer EdU-positive cells were noted among *Acp5*^*−/−*^ PMLFs than among WT PMLFs (Fig. 3e). Consistently, the silencing of *ACP5* expression remarkably reduced the proliferation of fibroblast (Fig. 3f). Similar data were also detected in PMLFs and PHLFs by CFSE staining (Supplementary Fig. 2a, b). Furthermore, we examined the migration of fibroblast by Transwell assay. Notably, the loss of *ACP5* significantly suppressed the migration of PMLFs and PHLFs across the Transwell membrane (Fig. 3g, h).

### ACP5 interacts with β-catenin and regulates the degradation of β-catenin

**Fig. 4:**
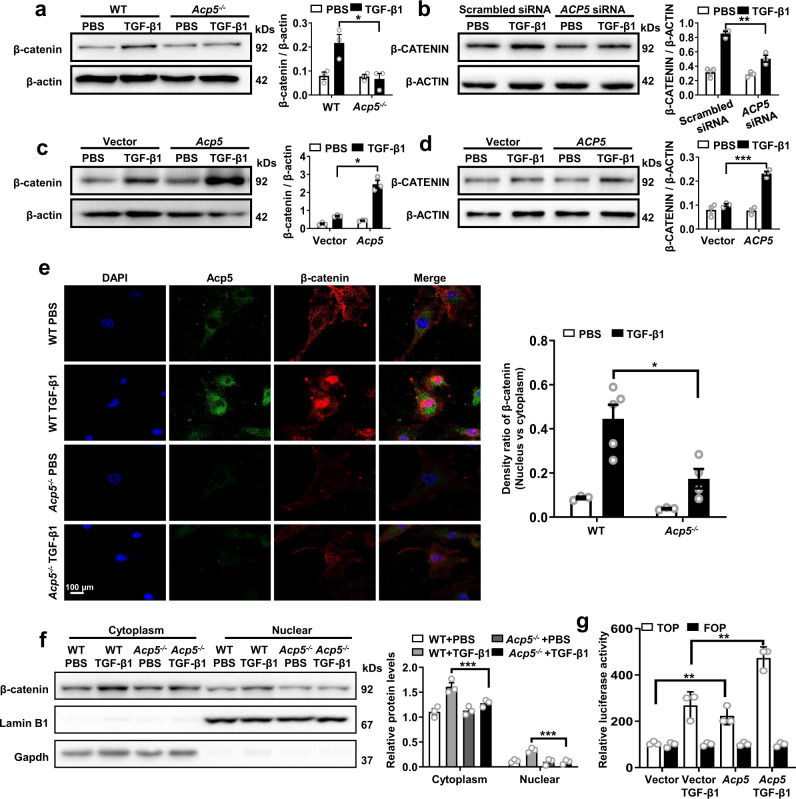
Altered Acp5 expression affects the levels of β-catenin. **a**–**d** Western blot analysis of the levels of β-catenin in WT or *Acp5*^*−/−*^ PMLFs (**a**, *p* = 0.0239), *ACP5* siRNA or Scrambled siRNA treated PHLFs (**b**, *p* = 0.0034), *Acp5-*plasmid or Vector treated *Acp5*^*−/−*^ PMLFs (**c**, *p* = 0.0138) and *ACP5-*plasmid or Vector treated PHLFs (**d**, *p* = 0.0003). **e** Representative results for coimmunostaining of Acp5 and β-catenin in PMLFs from WT and *Acp5*^*−/−*^ PMLFs following TGF-β1 induction (*p* = 0.0127). The nuclei were stained blue by DAPI, and the images were taken under original magnification ×400. **f** Western blot analysis of the levels of β-catenin in cytoplasm (*p* < 0.0001) and nuclear (*p* = 0.0001). **g** Normalized luciferase activities of TOP-Flash over FOP-Flash relative renilla luciferase units (RLU) in PMLFs (Vector treated versus *Acp5*-plasmid treated: *p* = 0.0064, Vector treated with TGF-β1 versus *Acp5*-plasmid treated with TGF-β1: *p* = 0.0094). The data are represented as the mean ± SEM of three independent experiments. Two-sided Student’s *t*-test (**a**–**b, d**–**g**) and two-sided unpaired Student’s *t*-test with Welch’s correction (**c**) were applied. *p < 0.05; **p < 0.01; ***p < 0.001. Source data are provided as a Source Data file.

β-catenin signaling is known to be critical in the process of pulmonary fibrosis^22^. Therefore, we investigated the impact of Acp5 on β-catenin signaling in TGF-β1-stimulated fibroblasts. As expected, high levels of β-catenin were detected in WT PMLFs after 24 h of TGF-β1 treatment. However, the protein but not mRNA levels of β-catenin were much lower in *Acp5*^*−/−*^ PMLFs, indicating that the decrease in β-catenin levels was not due to decreased gene transcription (Fig. 4a and Supplementary Fig. 3a). Consistently, the knockdown of *ACP5* expression in PHLFs also revealed lower levels of β-CATENIN following TGF-β1 induction compared to those in the group transfected with Scrambled siRNA (Fig. 4b). To confirm these results, the expression of *ACP5* was enhanced in *Acp5*^*−/−*^ PMLFs and PHLFs by plasmid transfection. In contrast, a remarkable increase in β-CATENIN was observed in both PMLFs and PHLFs after *ACP5-*plasmid treated followed by TGF-β1 stimulation (Fig. 4c, d). Nevertheless, the mRNA levels of *β-CATENIN* were also not affected by *ACP5* overexpression (Supplementary Fig. 3b).

Given that β-catenin translocation into the nucleus is critical for its function^23^, we next estimated the localization of Acp5 and β-catenin in PMLFs. Immunofluorescence assays revealed that both the Acp5 and β-catenin were mainly localized to the cytoplasm before treatment, while β-catenin translocation into the nucleus upon TGF-β1 stimulation was noted, and high expression of Acp5 was noted in the cytoplasm. However, much lower levels of β-catenin were detected in *Acp5*^*−/−*^ PMLFs than in WT PMLFs (Fig. 4e). Consistently, Acp5 was likely to increase the levels of β-catenin, as more *Acp5*^+^/β-catenin^+^ cells were detected in the lung section from BLM-induced mice compared with saline-treated mice (Supplementary Fig. 4). In contrast, *Acp5* deficiency evidently blunted the increasing of β-catenin induced by BLM (Supplementary Fig. 4).

To further quantify the subcellular transfer of β-catenin, we utilized Western blot assay. Indeed, significantly higher levels of β-catenin were detected in cytoplasmic and nuclear proteins from WT PMLFs than in those from *Acp5*^*−/−*^ PMLFs (Fig. 4f). Next, we conducted TOP Flash/FOP Flash reporter experiments to assess the effect of Acp5 on β-catenin signaling. Notedly, we discovered that overexpression of Acp5 remarkably increases luciferase activity in TOP over FOP (Fig. 4g), indicating that Acp5 could positively regulate the activity of β-catenin.

**Fig. 5:**
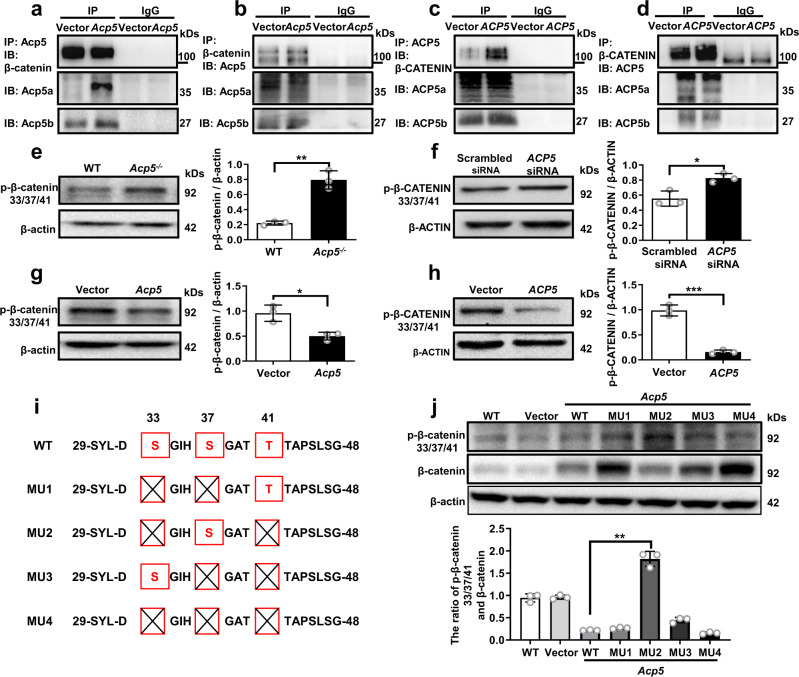
Acp5 dephosphorylates β-catenin at Ser33 and Thr41. **a**–**d** Coimmunoprecipitation of Acp5 and β-catenin in PMLFs (**a**, **b**) and in PHMLs (**c**, **d**). **e**–**h** Western blot analysis of the levels of p-β-catenin (S33, S37 and T41) in WT and *Acp5*^*−/−*^ PMLFs (**e**, *p* = 0.0013), *ACP5* siRNA and Scrambled siRNA treated PHLFs (**f**, *p* = 0.0159), *Acp5*-plasmid and Vector treated *Acp5*^*−/−*^ PMLFs (**g**, *p* = 0.0188) and *Acp5*-plasmid and Vector treated PHLFs (**h**, *p* = 0.0003). **i** The schematic results showing the mutant plasmids (MU1-3) of these three phosphorylated sites. Each of mutant plasmids (MU1-3) of these three phosphorylated sites maintained one normal site and two mutant sites (red boxes), and all phosphorylated sites were deleted in MU4. **j** Western blot analysis of the levels of β-catenin and p-β-catenin (S33, S37 and T41) in PMLFs following plasmids transduced (*p* = 0.0036). The data are represented as the mean ± SEM of three independent experiments. Two-sided Student’s *t*-test (**a**–**i**) and two-sided unpaired Student’s *t*-test with Welch’s correction (j) were applied. *p < 0.05; **p < 0.01; ***p < 0.001. Source data are provided as a Source Data file.

As compelling evidence suggests that Acp5 is a phosphatase^18,19^, we hypothesized that Acp5 dephosphorylated some sites on p-β-catenin, such as the sites Ser33, Ser37 and Thr41, which are involved in the process of β-catenin degradation^24^. Interestingly, Acp5 could interact with β-catenin in PMLFs (Fig. 5a, b) and PHLFs (Fig. 5c, d), as shown by co-IP assay. Furthermore, we discovered higher levels of β-catenin phosphorylated at Ser33, Ser37 and Thr41 in *Acp5*^*−/−*^ PMLFs (Fig. 5e) and *ACP5*-silenced PHLFs (Fig. 5f) than in WT PMLFs and Scrambled RNA-transfected PHLFs, respectively. In contrast, the opposite was observed in *ACP5*-overexpressing fibroblasts (Fig. 5g, h), indicating that Acp5 appears to dephosphorylate the Ser33, Ser37 and Thr41 sites in p-β-catenin, by which it inhibits the process of degradation. To further confirm the exact sites for Acp5 dephosphorylation, we constructed 4 plasmids containing mutated *β-catenin* (MU1, MU2, MU3 and MU4) (Fig. 5i) and transfected the mutant *β-catenin-*plasmids and *Acp5*-plasmid into PMLFs, followed by TGF-β1 stimulation. Interestingly, low ratio of p-β-catenin (33/37/41) and β-catenin were noted in PMLFs transfected MU plasmids except for MU2 (Fig. 5j), suggesting that Acp5 specifically dephosphorylates the Ser33 and Thr41 sites on p-β-catenin and inhibits the degradation of β-catenin.

### Global deletion of *Acp5* protects mice from BLM-induced lung injury and fibrosis

**Fig. 6:**
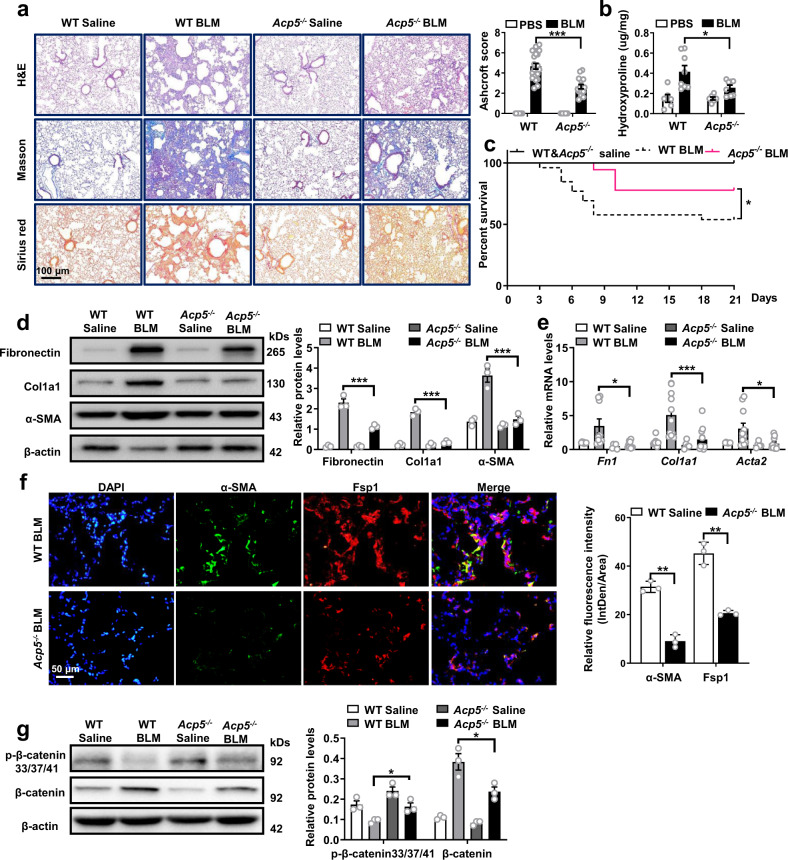
Comparison of the severity of lung fibrosis between WT and *Acp5*^*−/−*^ mice after BLM induction. **a** Histological analysis of the severity of lung fibrosis in mice after BLM induction. Left panel: representative images for H&E (top), Masson staining (middle) and Sirius red (bottom). Right panel: A bar graph showed the quantitative mean score of the severity of fibrosis (*p* < 0.0001). Images were captured at ×200 magnification (WT Saline *n* = 18, WT BLM *n* = 22, *Acp5*^*−/−*^ Saline *n* = 12, *Acp5*^*−/−*^ BLM *n* = 16). **b** Quantification of hydroxyproline contents in WT and *Acp5*^*−/−*^ mice (WT Saline *n* = 6, WT BLM *n* = 8, *Acp5*^*−/−*^ Saline *n* = 6, *Acp5*^*−/−*^ BLM *n* = 8, *p* = 0.0413). **c** The survival ratio in WT and *Acp5*^*−/−*^ mice after BLM induction (WT Saline *n* = 18, WT BLM *n* = 40, *Acp5*^*−/−*^ Saline *n* = 12, *Acp5*^*−/−*^ BLM *n* = 21, *p* = 0.0414). **d** Western blot analysis of Fibronectin (*p* < 0.0001), Col1a1 (*p* < 0.0001) and α-SMA (*p* < 0.0001) expression in the lung homogenate from WT (*n* = 3) and *Acp5*^*−/−*^ (*n* = 3) mice. **e** RT-PCR analysis of *Fn1* (*p* = 0.0269), *Col1a1* (*p* = 0.0001) and *Acta2* (*p* = 0.0214) expression in the lung homogenate from WT (n = 10) and *Acp5*^*−/−*^ (*n* = 11) mice. **f** Coimmunostaining of Fsp1 (*p* = 0.0025) and α-SMA (*p* = 0.0017) in the lung sections from WT (*n* = 3) and *Acp5*^*−/−*^ (*n* = 3) mice. The nuclei were stained blue by DAPI, and the images were taken under original magnification ×400. **g** Western blot analysis of the levels of β-catenin (*p* = 0.0350) and p-β-catenin (S33, S37 and T41, *p* = 0.0238) in WT (*n* = 3) and *Acp5*^*−/−*^ (*n* = 3) mice after BLM challenge. The data are represented as the mean ± SEM. Two-sided Student’s *t*-test (**a, c**–**g**) and two-sided unpaired Studen*t*’s *t*-test with Welch’s correction (**b, e**) were applied. *p < 0.05; **p < 0.01; ***p < 0.001. Source data are provided as a Source Data file.

To dissect the requirement of Acp5 in the development of lung fibrosis, *Acp5*^*−/−*^ and WT mice were subjected to BLM treatment. Subsequently, lung injury and fibrosis were assessed after 21 days of BLM induction. As expected, Hematoxylin and eosin (H&E), Masson’s trichrome and Sirius red staining of the lung sections from mice in the BLM group demonstrated a remarkable increase in lung parenchymal fibrotic lesions compared to those in the saline group (Fig. 6a). Notably, compared with *Acp5*^*−/−*^ mice, WT mice were more susceptible to bleomycin toxicity, as evidenced by higher Ashcroft scores (Fig. 6a), increased levels of hydroxyproline (Fig. 6b) and lower survival rates (Fig. 6c). To further quantitatively determine whether the loss of *Acp5* could inhibit fibrotic markers expression in the lungs of BLM-treated mice, we evaluated the protein and mRNA expression levels of fibrotic genes by Western blot and RT-PCR, respectively. As illustrated in Fig. 6d, e, the protein and mRNA levels of Fibronectin, Col1a1, and α-SMA after BLM treatment were significantly lower in the *Acp5*^*−/−*^ mice than in the WT mice. Additionally, *Acp5* deficiency remarkably attenuated the transition of fibroblast to myofibroblast, as evidenced by decreased fluorescence intensity for Fsp1^+^/α-SMA^+^ cells in the fibrotic lesion (Fig. 6f). Consistent with the effect of Acp5 on fibroblast proliferation in vitro, staining for Pcna and Fsp1 in the adjacent lung sections showed the decreased ability of fibroblast in *Acp5*^*−/−*^ mice to proliferate (Supplementary Fig. 5). Furthermore, the levels of β-catenin and p-β-catenin were also detected in WT and *Acp5*^*−/−*^ mice following BLM induction. Consistently, the levels of p-β-catenin were decreased and the levels of β-catenin were increased in the lung homogenates derived from the BLM-induced mice compared to control mice (Fig. 6g), and loss of *Acp5* largely reversed changes in the levels of β-catenin and p-β-catenin induced by BLM (Fig. 6g). Collectively, our data demonstrate that the loss of *Acp5* protects mice against BLM-induced lung injury and fibrosis.

### In vivo treatment with liposomes carrying *Acp5* siRNA evokes an antifibrotic response in BLM-induced pulmonary fibrosis

**Fig. 7:**
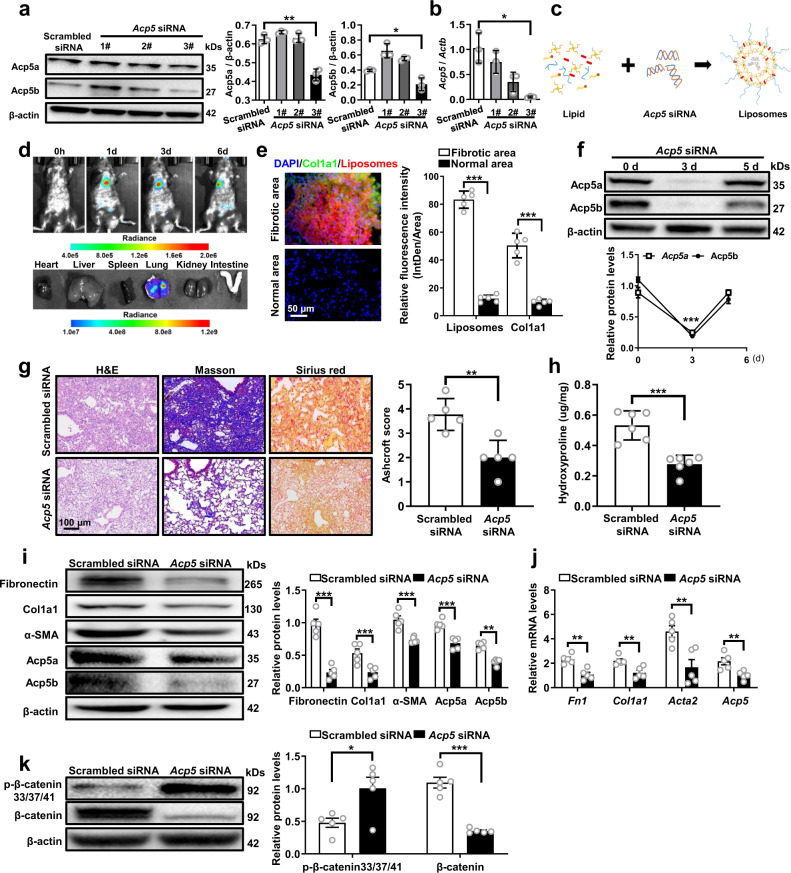
Administration of *Acp5* siRNA-loaded liposomes protected mice from BLM-induced lung injury and fibrosis. **a**, **b** Western blot (**a**, Acp5a: *p* = 0.0011, Acp5b: *p* = 0.0205) and RT-PCR (**b**, *p* = 0.0300) analysis of the interfering efficiency of *Acp5* siRNAs in PMLFs (*n* = 3 for each group). **c** Schematic diagram for preparation of liposomes carrying *Acp5* siRNA. **d** The biodistribution of the liposomes in pulmonary fibrosis model mice (*n* = 6). **e** Representative images of immunofluorescence for the biodistribution of liposomes (Red, *p* < 0.0001) and Col1a1 (Green, *p* = 0.0003) in lungs from BLM-induced mice (*n* = 6). The nuclei were stained blue by DAPI, and the images were taken under original magnification ×400. **f** Temporal Acp5 expression changes in lungs from transfected mice (*n* = 5, *p* < 0.0001). **g** Histological analysis of the severity of lung fibrosis in mice after BLM induction (Scrambled siRNA liposomes group *n* = 5, *Acp5* siRNA liposomes group *n* = 5, *p* = 0.0034). Images were captured at ×200 magnification. **h** Quantification of hydroxyproline contents in Scrambled or *Acp5* siRNA-loaded liposomes treated mice (*n* = 5) after BLM injection (*p* = 0.0002). **i**–**j**: Western blot (**i**) and RT-PCR (**j**) analysis of Fibronectin (**i**: *p* < 0.0001, **j**: *p* = 0.0012), Col1a1 (**i**: *p* = 0.0003, **j**: *p* = 0.0057), α-SMA (**i**: *p* = 0.0001, **j**: *p* = 0.0061), Acp5a (**i**: *p* = 0.0004), Acp5b (**i**: *p* = 0.0023) and *Acp5* (*p* = 0.0083) expression in the lung homogenate from Scrambled or *Acp5* siRNA-loaded liposomes treated mice (*n* = 5). **k** Western blot analysis of the levels of β-catenin (*p* = 0.0007) and p-β-catenin (S33, S37 and T41, *p* = 0.0202) in Scrambled or *Acp5* siRNA-loaded liposomes treated mice (*n* = 5). The data are represented as the mean ± SEM. Two-sided Student’s *t* test (**a, e**–**k**) and two-sided unpaired Student’s *t*-test with Welch’s correction (**b, e, k**) were applied. *p < 0.05; **p < 0.01; ***p < 0.001. Source data are provided as a Source Data file.

We sought to transform the above discoveries into a therapeutic approach to remedy pulmonary fibrosis. First, we verified the knockdown efficiency of three siRNAs in PMLFs (Fig. 7a, b) and chose the most efficient siRNA (#3) to generate lipid-based nanoparticles loaded with *Acp5* siRNA (Fig. 7c). The prepared liposomes demonstrated a > 90% entrapment efficiency for loading siRNA with a zeta potential of 4.1 mV (Supplementary Fig. 6a). Additionally, as observed in the transmission electron microscopy (TEM) images (Supplementary Fig. 6b), the prepared liposomes showed an average diameter of ~100 nm and a uniform sphere morphology. Furthermore, those liposomes illustrated a normal hydrodynamic diameter distribution (Supplementary Fig. 6c) and were continuously stable over 24 h (Supplementary Fig. 6d). To address the biodistribution of the liposomes in the lung, DiR-labeled liposomes were prepared. After intratracheal injection of the liposomes, mice were imaged by IVIS at different time points (0 h, 1d, 3d, 6d). The fluorescence signal of liposomes was mainly concentrated in the lung and gradually decreased over time (Fig. 7d). Additionally, similar results were obtained in ex vivo images (Fig. 7d). To further investigate the cellular localization of the liposomes in the fibrotic lung, pulmonary slides were stained with immunofluorescence. Interestingly, most of the fibroblasts overlapped the liposomes, which were mainly located in the fibrotic area, revealing the highly efficient absorption of fibroblasts (Fig. 7e). Then, the temporal expression of Acp5 was assessed after the delivery of the liposomes. Notably, a significant decline in Acp5a and Acp5b were noted, and the lowest Acp5a and Acp5b expression were detected at day 3 after the intratracheal delivery of *Acp5* siRNA-loaded liposomes; while, Acp5 expression was restored to normal levels at day 5 (Fig. 7f).

Ultimately, the WT mice were administered Scrambled siRNA- or *Acp5* siRNA-loaded liposomes on day 14 and day 18, respectively, by intratracheal instillation (1 mg/kg). Indeed, the therapeutic effects of siRNA-loaded liposomes were validated in BLM-induced mice, as manifested by the results of histopathological analysis (Fig. 7g), fibrotic scores (Fig. 7g) and decreased levels of hydroxyproline in the lung (Fig. 7h). Consistently, mice administered *Acp5* siRNA-loaded liposomes exhibited marked attenuation of fibrotic markers (Fibronectin, Col1a1, α-SMA) at the protein level (Fig. 7i) and mRNA level (Fig. 7j). Additionally, high levels of p-β-catenin (33/37/41) and low levels of β-catenin were noted in *Acp5* siRNA-loaded liposomes treated mice (Fig. 7k). Collectively, our experiments reveal the clinical potential of intratracheal *Acp5* siRNA-loaded liposomes administration in the treatment of pulmonary fibrosis.

### Administration of AubipyOMe reverses the established pulmonary fibrosis

**Fig. 8:**
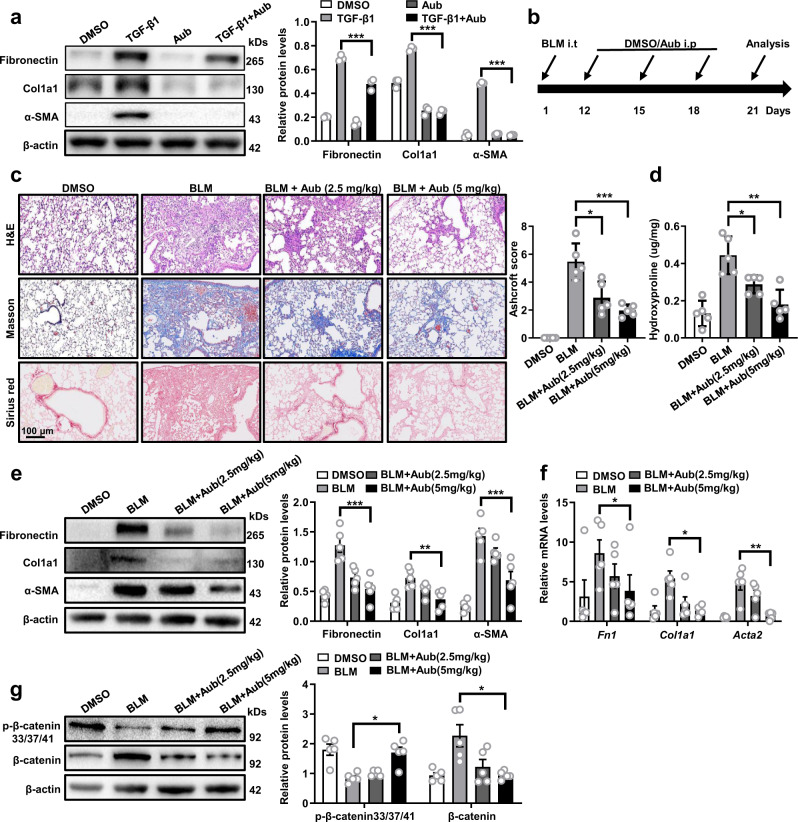
Treatment with AubipyOMe (Aub) reversed the established pulmonary fibrosis. **a** Western blot analysis of Fibronectin (*p* < 0.0001), Col1a1 (*p* < 0.0001) and α-SMA (*p* < 0.0001) in AubipyOMe treated PMLFs after TGF-β1 stimulation, Dimethyl Sulfoxide (DMSO) treated as control group. **b** Schematic diagram for BLM-induced model of pulmonary fibrosis and the AubipyOMe-delivery method (i.t: Intratracheal instillation, i.p: intraperitonea). **c** Histological analysis of the severity of lung fibrosis in mice after BLM induction. Left panel: representative images for H&E (top), Masson staining (middle) and Sirius red (bottom). Right panel: A bar graph showed the quantitative mean score of the severity of fibrosis. Images were captured at ×200 magnification. (BLM versus BLM with 2.5 mg/kg AubipyOMe: *p* = 0.0110, BLM versus BLM with 5.0 mg/kg AubipyOMe: *p* = 0.0005) **d** Quantification of hydroxyproline contents. (BLM versus BLM with 2.5 mg/kg AubipyOMe: *p* = 0.0147, BLM versus BLM with 5.0 mg/kg AubipyOMe: *p* = 0.0018) **e-f** Western blot (**e**) and RT-PCR (**f**) analysis of Fibronectin (**e**: *p* < 0.0001, **f**: *p* = 0.0146), Col1a1 (**e**: *p* = 0.0066, **f**: *p* = 0.0101), and α-SMA (**e**: *p* < 0.0001, **f**: *p* = 0.0083) expression. **g** W**e**stern blot analysis of the levels of β-catenin (*p* = 0.02241) and p-β-catenin (S33, S37 and T41, *p* = 0.0213). Each bar represents the mean ± SEM of 5 mice analyzed. Two-sided Student’s *t* test (**a**–**g**) and two-sided unpaired Student’s *t*-test wi*t*h Welch’s correction (**f**, **g**) were applied. *p < 0.05; **p < 0.01; ***p < 0.001. Source data are provided as a Source Data file.

To further illustrate the therapeutic effects of targeting Acp5 on pulmonary fibrosis, we applied an Acp5 inhibitor, AubipyOMe. Consistently, treatment of the PMLFs with AubipyOMe (100 nM) remarkably alleviated fibroblast to myofibroblast transition induced by TGF-β1 (Fig. 8a). Based on these observations, we next induced pulmonary fibrosis as above, and intraperitoneally injected the mice with different dose of AubipyOMe (2.5 mg/kg and 5 mg/kg) on days 12, 15, and 18 (Fig. 8b). Strikingly attenuated lung injury and fibrosis were detected in AubipyOMe treated mice, as shown by the H&E, Masson’s trichrome and Sirius red staining (Fig. 8c). In particular, the AubipyOMe- (5 mg/kg) treated mice achieved a lower Ashcroft score than the AubipyOMe- (2.5 mg/kg) treated mice (Fig. 8c), suggesting AubipyOMe reversed established pulmonary fibrosis in a dose depend manner. Additionally, similar results were obtained in the levels of hydroxyproline (Fig. 8d). Consistently, mice administered AubipyOMe exhibited marked attenuation of fibrotic markers at the protein level (Fig. 8e) and mRNA level (Fig. 8f). Furthermore, treatment with AubipyOMe significantly diminished the levels of β-catenin, along with increasing p-β-catenin (33/37/41) (Fig. 8g). More excitingly, AubipyOMe did not seem to have toxic effects on mice, as we failed to detect perceptible differences in the pathological staining of heart, liver, spleen, kidney and intestinal tract or biochemical indexes of liver, myocardium and kidney, signifying that AubipyOMe is well tolerated and safe for the treatment of pulmonary fibrosis (Supplementary Fig. 7). Collectively, our data provide compelling evidence demonstrating the therapeutic effects of AubipyOMe on pulmonary fibrosis.

## Discussion

**Fig. 9:**
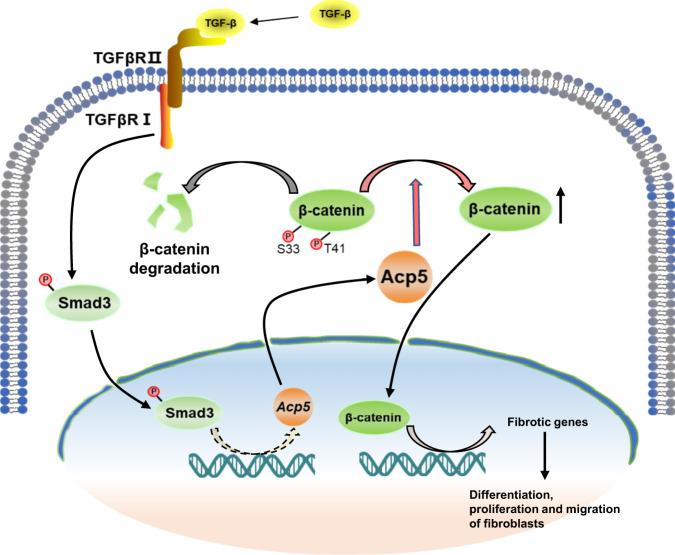
Schematic illustration of the mechanisms of *Acp5* in fibroblasts. *Acp5* is upregulated by TGF-β1 in a TGFβR1/Smad3 depend manner, and then *Acp5* specially binds to p-β-catenin and dephosphorylate the sites of Ser33 and Thr41, by which it resists the degradation of β-catenin and enhanced β-catenin signaling in the nuclear to promote the differentiation, proliferation and migration of fibroblasts.

As outcomes in pulmonary fibrosis, especially IPF, remain poor despite the emergence of some new antifibrotic agents, an improved understanding of the factors that influence gene expression and fibroblast differentiation is required. Although ACP5 has extensive pathophysiological functions depending on the cell type and disease^25^, our study provides direct experimental proof that ACP5 is involved in the pathogenesis of pulmonary fibrosis. We detected an increase in the levels of ACP5 within IPF samples compared to the control subjects. Remarkably, higher levels of ACP5 were likely noted in the sera of IPF patients with lower DLCO predicted values. Furthermore, *Acp5* deficiency protected mice from BLM-induced lung injury and fibrosis, with decreases in the differentiation of fibroblast to myofibroblast. Mechanistic experiments revealed that Acp5 was upregulated by TGF-β1 in a TGFβR1/Smad3-dependent manner, after which Acp5 specifically bound to p-β-catenin and dephosphorylated the sites Ser33 and Thr41, by which it inhibited the degradation of β-catenin and enhanced β-catenin signaling in the nucleus to promote the differentiation, proliferation and migration of fibroblast (Fig. 9). Notably, the treatment of mice with *Acp5* siRNA-loaded liposomes or Acp5 inhibitor strikingly reversed the pulmonary fibrosis. Taken together, our results not only provide insights into the understanding of IPF pathogenesis but also strongly suggest that strategies aimed at silencing or suppressing ACP5 could be viable approaches for the treatment of pulmonary fibrosis in clinical settings.

There is compelling evidence that ACP5, as a pivotal mammalian member, is a kind of generally distributed and well-conserved protein^25^. ACP5 exists in two isoforms: ACP5a is a monomer with the molecular weight as 35kD, while ACP5b is a dimer (molecular weight are 16kD and 23kD, respectively) derived from ACP5a by proteolytic cleavage of a repressive loop domain^25,26^. Notedly, a raised expression of ACP5 is detected in many pathological conditions^25^. Specifically, ACP5 is upregulated in the progression of plentiful types of tumors such as breast cancer^18^, lung cancer^19^, ovarian cancer^27^, malignant melanoma^28^ and hepatocellular carcinoma^29^. Furthermore, ACP5 is used as a biochemical maker for evaluating the bone metastasis of various tumors^30^. As the pathways and risk factors are often shared by cancer and IPF^31,32^, we detected the levels of ACP5 in sera and lung samples from IPF patients. Interestingly, high levels of ACP5 were detected in sera and lung sections from IPF patients. The exciting discovery in this report was that the serum concentration of ACP5 was likely associated with decreased lung function in IPF patients. Specifically, higher levels of ACP5 were likely observed in patients with severe IPF (DLCO < 40% predicted), indicating that ACP5 may be a surrogate biochemical marker for the severity of IPF.

We next focused on assessing cells that showed aberrant ACP5 expression in lung sections from IPF patients. Interestingly, we illustrated that ACP5 was predominantly localized within α-SMA^+^ myofibroblasts in IPF. Similarly, Acp5 was noted to be overexpressed in myofibroblasts derived from mice with BLM-induced pulmonary fibrosis. Additionally, the expression of ACP5 was also detected in PMLFs and PHLFs following TGF-β1 treatment. Furthermore, we showed that Acp5 was expressed in myofibroblasts in a TGFβR1- and Smad3-dependent manner, as manifested by the detection of decreased levels of Acp5 in fibroblasts treated with SB431542 or SIS3-HCL followed by TGF-β1 stimulation. Myofibroblasts are critical in the process of pulmonary fibrosis by their secretion of ECM proteins, leading to tissue stiffness and respiratory failure^33^. A recent study has demonstrated that myofibroblasts in fibrotic lungs are mainly derived from resident lung fibroblasts^10^. This discovery prompted us to assess the impact of Acp5 on fibroblasts. Deficiency, silence or inhibition of *Acp5* significantly abolished the differentiation, proliferation and migration of fibroblasts, respectively. As a result, mice exhibited a significant reduction in BLM-induced pulmonary fibrosis after suppressing Acp5 function.

Given that macrophages express of ACP5^34^ and alternative activated macrophages (M2), producing abundant of fibrotic cytokines, play a critical role in the pathogenesis of IPF^35,36^, we dissected the effects of Acp5 on macrophages activation. Interestingly, the similar number of CD68^+^Acp5^+^ cells was detected in the lung sections from saline and BLM-treated mice (Supplementary Fig. 8a). Consistently, neither perceptible difference was noted in the expression of *Acp5* (Supplementary Fig. 8b), nor AubipyOMe affect the polarization of M2 macrophages (Supplementary Fig. 8c). Although depleting the macrophage in the lung, knockdown the expression of *Acp5* still protected the mice from BLM-induced pulmonary fibrosis (Supplementary Fig. 8d–g), indicating that the protection effects of *Acp5* silencing were likely from inhibition of the fibroblast transition, not macrophage polarization.

The next important issue was how Acp5 expression promoted the differentiation, proliferation and migration of fibroblast. Previous studies have illustrated that aberrant β-catenin activation is involved in the pathogenesis of IPF^22,37^. In addition, β-catenin signaling is required for TGF-β-induced fibroblast differentiation, proliferation and migration^38,39^. We therefore examined the effects of Acp5 on β-catenin signaling in fibroblasts. Notedly, the loss or knockdown of *Acp5* robustly decreased the levels of β-catenin in the cytoplasm and nucleus. Conversely, more β-catenin was observed in *ACP5-*plasmid-transfected fibroblasts than the controls. It was likely that the impact of *Acp5* on β-catenin was not regulated at the transcriptional level, as β-catenin mRNA was not altered in *Acp5*-knockdown or *Acp5*-overexpression fibroblasts. A previous study showed that cytoplasmic β-catenin in the destruction complex was phosphorylated at residues Ser33, Ser37, and Thr41 in the absence of Wnt ligands; the p-β-catenin was recognized by the E3 ubiquitin ligase β-transducin repeat-containing protein (β-TrCP) and subsequently degraded by the ubiquitin-dependent proteasome pathway^40^. Indeed, high levels of p-β-catenin (Ser33, Ser37, and Thr41) were observed in *Acp5*-deficient or *Acp5*-silenced fibroblasts. Accumulating evidence demonstrated that ACP5 was a multifunctional protein. In physiological setting, ACP5 is secreted into the resorption region of osteoclasts and dephosphorylates bone matrix phosphoproteins, maintaining the balance of bone metabolism^41^. In recent years, ACP5 has been discovered as a plasmosin and is responsible for the dephosphorylation of Man6P-containing lysosomal proteins^42^ and P53^19^. Consistently, in this study, we identified p-β-catenin as a substrate for Acp5. According to the previous studies^19,25,26^, ACP5b exhibited much more phosphatase activity than ACP5a. Therefore, we speculated it was likely ACP5b, other than ACP5a, bound to p-β-catenin and then dephosphorylated the Ser33 and Thr41 sites, subsequently inhibiting the degradation of β-catenin.

Given that until now, no effective therapies to halt the progression of IPF have been available, we assessed the efficacy of targeting Acp5 in mice with fibrosis induced by BLM. Previous studies have established liposomes as drug carriers for inhalation owing to their safety and ability to provide controlled drug release in the lung^43,44^. In 2018, the first siRNA loaded liposomes (patisiran) was approved by the FDA, which moved gene therapy from concept to clinical application^45^. Similarly, in our study, *Acp5* siRNA-loaded liposomes were efficiently taken up by fibroblasts in lung lesions after intratracheal injection. Notably, liposomes carrying *Acp5* siRNA significantly attenuated lung fibrosis during the “fibrotic” phase of the model, which is more applicable to the clinical management of IPF patients. Simultaneously, we also assessed the therapeutic effects of the Acp5 inhibitor, AubipyOMe. More excitingly, AubipyOMe evidently reversed established pulmonary fibrosis in a dose depend manner without toxic effects on mice, further suggesting the therapeutic potential of targeting Acp5 on pulmonary fibrosis in clinical practice.

Our study has some limitations. First, the numbers of the IPF cohort were small, even if statistically significant was noted, more IPF patients are needed to verify the levels of ACP5 in the sera and explore the cellular source. Second, although we demonstrated that Acp5 specifically bound to p-β-catenin and dephosphorylated the sites Ser33 and Thr41, until now, no commercial antibodies for p-β-catenin (Ser33), p-β-catenin (Thr41), or p-β-catenin (Ser33 and Thr41), except for commercial antibodies for p-β-catenin (Ser33, Ser37, and Thr41), have been available. Third, we illustrated that Acp5 was upregulated by TGF-β1 in a TGFβR1/Smad3-dependent manner, but the detailed mechanism needs to be further explored. Finally, it is likely that deficiency of *Acp5* protects the mice from pulmonary fibrosis mainly via attenuating fibroblast to myofibroblast transition. However, further studies are needed to fully elucidate the effects of Acp5 on other cells function in the pathogenesis of IPF.

In conclusion, this report demonstrates that ACP5 is involved in the progression of pulmonary fibrosis. High serum levels of ACP5 are likely associated with the severity of IPF patients. In addition, mice with *Acp5* deficiency were protected from BLM-induced lung injury and fibrosis coupled with a significant reduction of fibroblast differentiation. Mechanistic experiments show that Acp5 is upregulated by TGF-β1 in a TGFβR1/Smad3-dependent manner, after which Acp5 selectively dephosphorylates p-β-catenin at Ser33 and Thr41 in the cytoplasm, reducing the degradation of β-catenin, by which the levels of β-catenin in the nucleus are enhanced, promoting fibroblast differentiation, proliferation and migration. Together, our data indicate that targeting Acp5 may represent a promising therapeutic approach for the treatment of pulmonary fibrosis in clinical settings.

## Methods

### Human samples

Sera from patients with IPF (*n* = 20) and control subjects (*n* = 13), lung explant material from IPF patients (*n* = 5) and resected para-carcinoma lung tissues from nonsmall-cell lung cancer (NSCLC) patients (*n* = 5) were collected at Tongji Hospital. Written informed consent was obtained from all participants. An IPF diagnosis was made according to consensus diagnostic criteria from the European Respiratory Society (ERS)/American Thoracic Society (ATS)^46^. The whole study was conducted in compliance with the Declaration of Helsinki and approved by the Human Assurance Committee of Tongji Hospital. Clinical data from IPF patients and control subjects were provided in Supplementary Table 1.

### Measurement of serum ACP5 levels

To detect ACP5 in the sera of IPF patients and control subjects, an ACP5 ELISA kit (Hycult Biotechnology, Uden, The Netherlands) was used in accordance with the manufacturer’s protocol. Briefly, whole-blood samples originating from IPF patients and control subjects were centrifuged at 900 x *g* for 15 min. Each sample was assayed in duplicate. ELISA plates were scanned on a microtiter plate reader (ELx800, BioTek Instruments, Inc., Winooski, VT) at 450 nm. Levels of ACP5 were calculated according to a standard curve.

### Immunofluorescence staining

Cryosections of lung tissues from IPF patients and mice with the onset of BLM-induced pulmonary fibrosis were used for immunofluorescence staining. The primary antibodies used for staining were as follows: mouse anti-ACP5 (Abnova, Taipei, China, 1: 100), rabbit anti-ACP5 (Proteintech, Wuhan, China, 1:100), rabbit anti-FSP1 (Proteintech, Wuhan, China, 1:100), mouse anti-α-SMA (Cell Signaling Technology, MA, USA, 1:100), rabbit anti-β-catenin (Cell Signaling Technology, MA, USA, 1:100), and rabbit antiphospho-Smad3 (Cell Signaling Technology, MA, USA, 1:100). Alexa 488- or 594-conjugated anti-mouse or rabbit (Abbkine, CA, USA, 1:400) were used as fluorescent secondary antibodies, and nuclei were counterstained with 4′-6-diamidino-2-phenylindole (DAPI). Images were obtained with a fluorescence microscope (Olympus, Shinjuku, Japan).

### Animals

*Acp5*-knockout mice (*Acp5*^*−/−*^ mice, C57BL/6 background) and wild-type mice (WT, C57BL/6 background) were purchased from GemPharmatech Co., Ltd. (Nanjing, China). *Acp5* depletion was confirmed by genotyping of the tail blood DNA (Supplementary Fig. 9). Genotyping of *Acp5*^*−/−*^ mice was performed using the following primers: primers 1: forward primer, 5′-TGC TAC TGG TGT GTC TGT GGA ACT G-3′ and reverse primer, 5′- TCA TGT TCA GCA GGA CCT TGC TAA-3′ and primers 2: forward primer, 5′- TGC TAC TGG TGT GTC TGT GGA ACT G-3′ and reverse primer, 5′- GAT CTC TTT GGC ATT GGC CAT TT-3′. All animals were housed in a specific pathogen-free animal facility at Tongji Hospital under a 12:12 h light/dark photocycle and provided food and water ad libitum. Animal experiments were conducted in accordance with all the relevant ethical guidelines. All experimental procedures were approved by the Animal Care and Use Committee at Tongji Hospital (TJH-201812005).

### BLM-mediated induction of pulmonary fibrosis

Pulmonary fibrosis was induced in male WT and *Acp5*^*−/−*^ mice (8–10 weeks old). Briefly, the mice were anesthetized with 1% pentobarbital sodium (60 mg/kg) and then intratracheally administered 2 U/kg BLM (Huirui, Shanghai, China) in 40 μl of sterile saline with a high pressure atomizing needle (Cat: BJ-PW-M, Bio Jane Trading Limited, Shanghai, China). Mice administered the same volume of sterile saline served as controls. SiRNA-loaded liposomes were injected into the anesthetized animals intratracheally on days 14 and 18 after BLM injection. AubipyOMe (Sigma-Aldrich, St. Louis, MO, USA) was intraperitoneally injected with different doses (2.5 mg/kg and 5 mg/kg) on days 12, 15, and 18 following BLM administration. Finally, the mice were euthanized on day 21 following the BLM challenge to analyze pulmonary fibrosis.

### Pathological staining and histopathologic assessment

The lungs were removed at 21 days after BLM or saline administration. Following fixation, the lungs were embedded in paraffin and sectioned. The sections were then stained with hematoxylin and eosin (H&E), Masson’s trichrome stain and Sirius red. Fibrosis was scored on a scale of 0–8 using the Ashcroft scoring method^47^. The severity of fibrotic changes in each histological section of the lung was assessed as the mean of the severity scores from the observed microscopic fields. Fifty randomly chosen regions from each mouse lung were graded, after which their scores were averaged, and the average scores are depicted in a graph at 200-fold magnification. Grading was performed in a blinded manner by three independent observers.

### Hydroxyproline assay

Lung collagen deposition was assessed by measuring the hydroxyproline content of lung homogenates with a hydroxyproline assay kit (BioVision, CA, USA) in accordance with the manufacturer’s protocol. Briefly, lung tissues were homogenized in ddH_2_O, after which an equal volume of concentrated 12 N HCl was added to the tissues in a pressure-tight, capped Teflon vial and hydrolyzed at 120 °C for 3 h. After centrifugation at 16000 x *g* for 3 min, 10 μl of each hydrolyzed sample was transferred to a 96-well plate. In each well, 100 μl of chloramine T reagent was added to the sample, which was incubated at room temperature for 5 min, after which 100 μl of DMAB reagent was added. After incubation for 90 min at 60 °C, the plates were read at 560 nm with a microtiter plate reader (ELx800, BioTek Instruments, Inc., Winooski, VT), and the hydroxyproline concentration in the sample was calculated from a standard curve and related to the amount of lung tissue used. The hydroxyproline contents in the lung tissues are given as μg of hydroxyproline per mg of lung tissue.

### Cell culture and treatment

PHLFs were isolated from para-carcinoma lung tissues resected from NSCLC patients, and PMLFs were isolated from the lung tissues of *Acp5*^*−/−*^ or WT mice^44^. Briefly, fibroblasts were generated by mincing lung tissue into submillimeter-sized pieces, plated evenly in 100 mm plates containing 2 ml of medium, which was changed after 24 h. Cells were cultured in DMEM containing 10% fetal bovine serum (FBS) and penicillin/streptomycin at 37 °C and tested for mycoplasma regularly. PHLFs and PMLFs between passages 3 and 5 were used.

### Cell transfection

Small interfering RNAs (siRNAs) specific for *ACP5* and a corresponding Scrambled siRNA were purchased from RiboBio (Guangzhou, China) and then transiently transfected into PHLFs using Lipofectamine 3000 (Invitrogen, Shanghai, China)^36^. Briefly, PHLFs were seeded in 6-well or 96-well plates at 24 h before transfection. SiRNA transfection was performed when the cells reached 50–60% confluence. Transfection efficiency was monitored by RT-PCR or Western blot at 48 h after transfection. The siRNA specific for *ACP5* targeted the following sequence in *ACP5* mRNA: 5′-GAC ACT ATG TGG CAA CTC A-3′.

*ACP5*-plasmid and a vector plasmid were purchased from GeneChem (Shanghai, China), and 2 μg of purified DNA was mixed with transfection reagent and applied to the cells. Forty-eight hours after transfection, the cells were analyzed by Western blot.

### Real-time PCR

Total RNA was isolated from lung tissues and fibroblasts with TRlzol reagent (Takara, Dalian, China), according to the manufacturers’ instructions. The RNA quantity and quality were measured using a NanoDrop 2000 spectrophotometer (Thermo Scientific, MA, USA). Complementary DNA synthesis was performed using an M-MLV reverse transcriptase kit (Invitrogen, CA, USA). Real-time PCR was performed on a CFX96. Real-time PCR detection system (Bio-Rad, CA, USA) using SYBR Green mix (Takara, Dalian, China) under the following conditions: 30 s at 95 °C for initial denaturation, followed by 40 cycles of 95 °C for 5 s and 60 °C for 30 s. The comparative Ct method was used to normalize the data for presentation as mean normalized transcript levels. The RNA levels of *Actb* were used to normalize the data. The primer sequences were provided in Supplementary Table 2.

### Western blot

Lung tissues and fibroblasts were homogenized in RIPA lysis buffer (Beyotime, Shanghai, China). For subcellular fractionation, the protein was extracted using NE-PER^TM^ Nuclear and Cytoplasmic Extraction reagents (Thermo Fisher Scientific, USA). Western blot was performed according to previously reported protocols^48^. The primary antibodies used were anti-ACP5 (Gentex, CA, USA, 1:1,000); anti-LAMIN B1, anti-FIBRONECTIN and anti-COL1A1 (Proteintech, Wuhan, China, 1:1,000); anti-α-SMA, antiphospho-β-CATENIN (Ser33/37/Thr41, Cell Signaling Technology, MA, USA, 1: 1000), and anti-β-CATENIN (Cell Signaling Technology, MA, USA, 1: 1000); and anti-β-ACTIN (Abcam, MA, USA, 1:3000). β-ACTIN bands were used as the loading control for cytoplasmic or total proteins, and LAMIN B1 bands were used as the loading control for nuclear proteins. Detection was performed using a chemiluminescent substrate system (Bio-Rad Laboratories, CA, USA). The gray values were analyzed with ImageJ software.

### Cell proliferation assay

PMLFs were cultured in 96-well plates at a density of 2 × 10^3^ cells/well. Cell proliferation was then measured using the EdU proliferation assay (Ribobio, Guangzhou, China), according to the manufacturers’ instructions. Briefly, 18 h after being seeded in the plates, cells were labeled with EdU for 2 h at 37 °C, treated with 100 µL of Apollo reaction cocktail and stained with 100 µL of Hoechst 33342. Finally, the cells were observed under a fluorescence microscope (Olympus, Shinjuku, Japan).

### Cell migration assay

Cell migration assays were performed using Transwell inserts with a membrane with a pore size of 8.0 μm (Corning, MA, USA) according to previously reported methods^49^. The cells (2.5 × 10^4^) were resuspended in 2% serum-containing medium and seeded into the upper chambers. The lower chambers were filled with complete culture medium containing 10% FBS to function as a chemoattractant. After incubation at 37 °C for 24 h, the cells migrated through the membrane filter and were stained with 0.1% crystal violet (Sigma-Aldrich, St. Louis, MO, USA).

### Coimmunoprecipitation assay

Immunoprecipitation was performed using whole-cell lysate according to a previous protocol^19^. Briefly, anti-ACP5 and anti-β-CATENIN antibodies were used to form immune complexes with the ACP5 and β-CATENIN proteins in lysates that were immunoprecipitated with magnetic beads (Cell Signaling Technology, Danvers, MA, USA) for 2 h at 4 °C. Then beads were washed three times with lysis buffer, and bound proteins were eluted in SDS sample buffer. Finally, equivalent samples were subjected to Western blot analysis for ACP5 and β-CATENIN.

### TOP/FOP-flash assay

TOP/FOP Flash (Genechem, Montreal, Quebec, Canada), plus *Acp5-*plasmid were co-transfected into PMLFs using the Lipofectamine 3000 reagent according to the manufacturers’ instructions. The luciferase activity of TOP Flash or FOP Flash was monitored via Dual Luciferase Report Assay System (Promega, Madison, WI, USA) according to a protocol provided by the manufacturer. The TOP/FOP ratio was then calculated to assess the activity of Wnt/β-catenin pathway.

### Immunohistochemistry

Immunohistochemistry (IHC) was performed on 3 μm adjacent lung sections. The primary antibodies used were anti-Pcna (Cell Signaling Technology, MA, USA, 1:100) and anti-Fsp1 (Proteintech, Wuhan, China, 1:100). The primary antibodies were incubated with the sections overnight at 4 °C, followed by incubation with a secondary antibody for 1 h at room temperature. Finally, the sections were stained with DAB reagent for 2 min at room temperature, and the nuclei were then counterstained with hematoxylin. Stained cells were observed under an optical photographic light microscope at ×400 magnification.

### Liposomes

SiRNA-loaded liposomes were prepared as reported^43^. A lipid solution in which the lipidoid, cholesterol, DSPC and mPEG-DMG at a molar ratio of 50:38.5:10:1.5 were dissolved in a solution of 90% ethanol and 10 mM sodium citrate was prepared. Then, siRNA was dissolved in 10 mM citrate buffer, and the lipid components were mixed with the dissolved siRNA by vortexing such that the final weight ratio of lipidoid:siRNA was 5:1. The next step was ultrafiltration centrifugation to exclude free siRNA. Finally, the siRNA-liposomes were diluted in PBS. The hydrodynamic diameter, polydispersity, zeta potential and stability of the liposomes were measured by dynamic light scattering (DLS) (Malvern Zetasizer Nano-ZS, UK). A RiboGreen assay was employed to calculate the siRNA entrapment efficiency. After staining with 2% phosphotungstic acid, the liposomes were characterized by transmission electron microscopy (TEM, Jeol, Japan).

### In vivo biodistribution of the liposomes

DiR-labelled liposomes were constructed as previous reported^44^, and then, the prepared liposomes were intratracheal injection into the mice after 14 days of BLM induction. Subsequently, the mice were anesthetized with isoflurane (Sigma-Aldrich, St. Louis, MO, USA) and photoed at different time points (0 h, 1d, 3d, 6d) by an in vivo imaging system (IVIS Lumina XR, SI Imaging, AZ, USA) (excitation: 745 nm, emission: 830 nm). After 6d, the mice were euthanized, and the organs were harvested for ex vivo fluorescence imaging, respectively.

### Statistical analyses

All statistical analyses were performed using GraphPad Prism (San Diego, CA, USA) or SPSS 25.0 (IBM, Armonk, NY, USA). Standard statistical analysis was applied to all the figures as appropriate and indicated in the figure legends. The data are presented as the mean ± SEM. In all cases, *p* < 0.05 was considered with statistical significance.

### Reporting summary

Further information on research design is available in the Nature Research Reporting Summary linked to this article.

## Supplementary information

Supplementary information

Reporting Summary

## Data Availability

The minimum dataset necessary to interpret, verify and extend the research in the article is provided in the manuscript, the supplementary information and the source data file. Any additional raw data related to the study will be available from the corresponding author upon reasonable request. Source data are provided with this paper.

## Source data

Source Data
