# Supplementary material for: Tartrate-resistant acid phosphatase 5 promotes pulmonary fibrosis by modulating β-catenin signaling
Source: Nat Commun. 2022 Jan 10;13:114. doi: 10.1038/s41467-021-27684-9 (PMC8748833; doi:10.1038/s41467-021-27684-9)
Supplement: Supplementary file 1 — Supplementary information [file 41467_2021_27684_MOESM1_ESM.pdf]

Supplementary information

Tartrate-resistant acid phosphatase 5 promotes pulmonary fibrosis by modulating β-catenin signaling

Yinan Hu[1,2,#], Qi Wang[1,#], Jun Yu[3,#], Qing Zhou[4], Yanhan Deng[1], Juan Liu[1], Lei Zhang[1], Yongjian Xu[1], Weining Xiong[1,5,*], Yi Wang[1,*]

[1]Department of Pulmonary and Critical Care Medicine, NHC Key Laboratory of Respiratory Diseases, Key Site of National Clinical Research Center for Respiratory Disease, Wuhan Clinical Medical Research Center for Chronic Airway Diseases, Tongji Hospital, Tongji Medical College, Huazhong University of Science and Technology, 1095 Jiefang Ave, Wuhan 430030, China.

[2]Department of Pulmonary and Critical Care Medicine, Center of Respiratory Medicine, National Center for Respiratory Medicine, Institute of Respiratory Medicine, Chinese Academy of Medical Sciences, National Clinical Research Center for Respiratory Diseases, China-Japan Friendship Hospital, 100029, Beijing, China.

[3]Department of Thoracic Surgery, Tongji Hospital, Tongji Medical College, Huazhong University of Science and Technology, 1095 Jiefang Ave, Wuhan 430030, China.

[4]The Center for Biomedical Research, Tongji Hospital, Tongji Medical College, Huazhong University of Science and Technology, 1095 Jiefang Ave, Wuhan 430030, China.

[5]Department of Pulmonary and Critical Care Medicine, Shanghai Key Laboratory of Tissue Engineering, Shanghai Ninth People's Hospital, Shanghai Jiaotong University School of Medicine, 639 Zhizaoju Lu, Shanghai, 200011, China.

[#]These authors contributed equally to this work.

*Correspondence: Yi Wang (Tel:86-27-83665521; E-mail: wangyi@tjh.tjmu.edu.cn), Department of Pulmonary and Critical Care Medicine, Tongji Hospital, Tongji Medical College, Huazhong University of Science and Technology; or Weining Xiong (Tel: 86-21-53316086; E-mail: xiongdoctor@qq.com), Department of Pulmonary and Critical Care Medicine, Shanghai Key Laboratory of Tissue Engineering, Shanghai Ninth People's Hospital, Shanghai Jiaotong University School of Medicine.

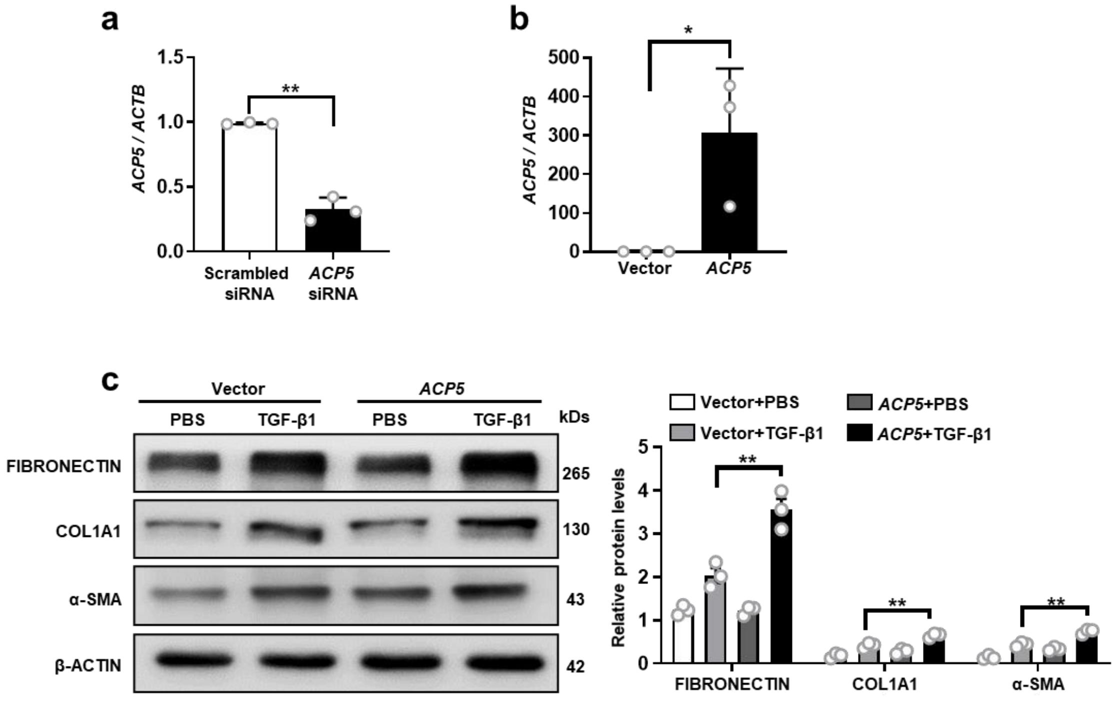

Supplementary Figure 1. Knockdown and overexpression efficiency of *ACP5* and the effects when following TGF-β1 induction. a-b RT-PCR analysis of the levels of *ACP5* in *ACP5* siRNA or Scrambled siRNA treated PHLFs (a, p = 0.0061) and *ACP5* plasmid or Vector treated PHLFs (**b**, p = 0.0335). **c** Western blot analysis of FIBRONECTIN (p = 0.0077), COL1A1 (p = 0.0075) and α-SMA (p = 0.0032) expression in *ACP5* plasmid or Vector treated PHLFs following TGF-β1 induction. The data are represented as the mean ± SEM of three independent experiments. Two-sided unpaired Student's *t*-test with Welch's correction (a, b) and two-sided Student's *t*-test (c) test was applied. *, $p < 0.05$; **, $p < 0.01$. Source data are provided as a Source Data file.

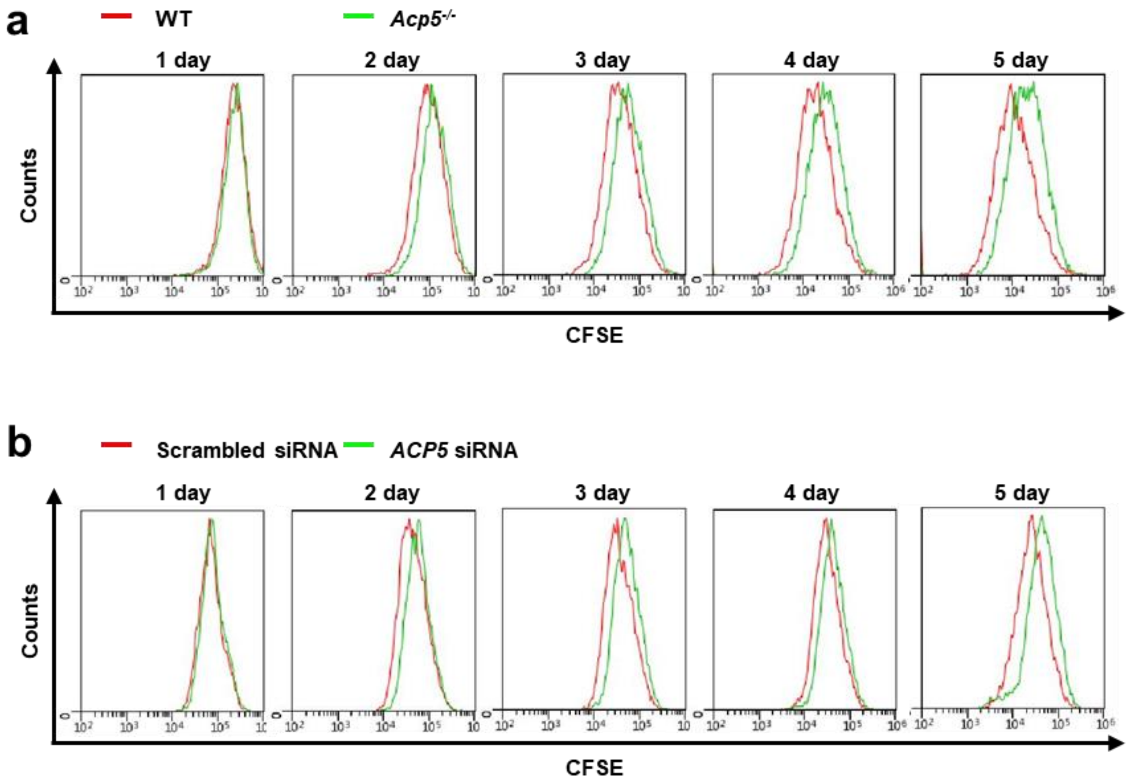

Supplementary Figure 2. CFSE staining analysis of proliferation. WT and *Acp5*$^{-/-}$ PMLFs (**a**), *ACP5* siRNA or Scrambled siRNA treated PHLFs (**b**). Source data are provided as a Source Data file.

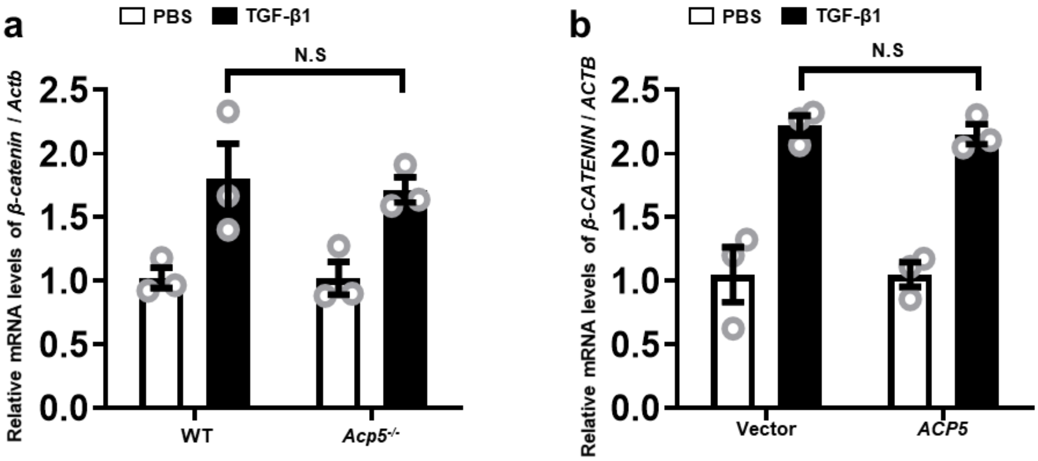

Supplementary Figure 3. The effects of *ACP5* on *β-catenin*. a-b RT-PCR analysis of the levels of *β-catenin* in WT and *Acp5*$^{-/-}$ PMLFs (**a,** p = 0.7841) and *ACP5* plasmid or Vector treated PHLFs (**b,** p =0.5723). The data are represented as the mean ± SEM of three independent experiments. Two-sided Student's *t*-test (a, b) test was applied. N.S, no significant difference between two groups. Source data are provided as a Source Data file.

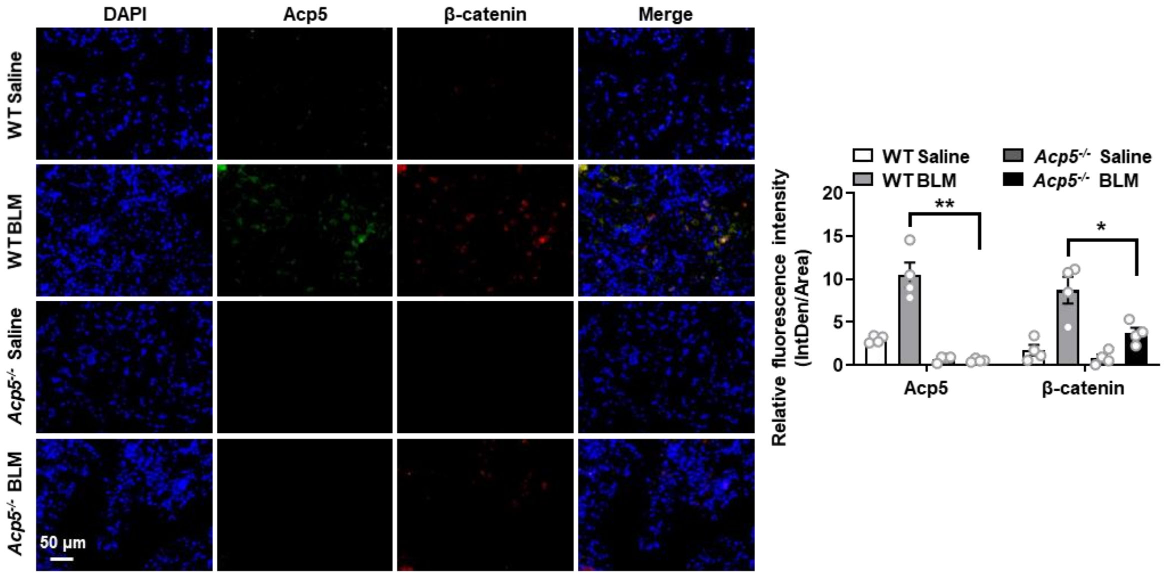

Supplementary Figure 4. Co-immunostaining of Acp5 and β-catenin. Representative results for co-immunostaining of Acp5 (p = 0.0064) and β-catenin (p = 0.0405) in the lung sections from WT and *Acp5*$^{-/-}$ mice following BLM injection. Each bar represents the mean ± SEM of 4 mice analyzed and two-sided unpaired Student's t-test with Welch's correction was applied. *, $p < 0.05$; **, $p < 0.01$. Source data are provided as a Source Data file.

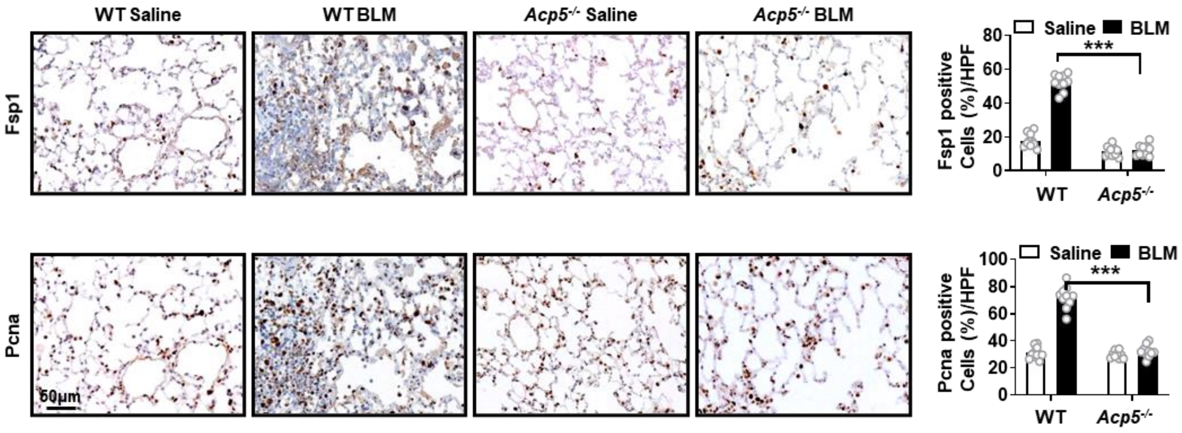

Supplementary Figure 5. IHC staining for proliferation. Representative IHC staining of adjacent lung tissue sections for Fsp1 ($p < 0.0001$) and Pcna ($p < 0.0001$). Ten mice were included in each study group. The data are represented as the mean ± SEM and an independent two-side Student's *t*-test was administered to analyze the statistical significance of differences between two groups. ***, $p < 0.001$. Source data are provided as a Source Data file.

a

	Blank liposome	siRNA loaded liposome
Hydrodynamic diameter (nm)	114	103
PDI	0.11	0.07
Zeta-potential (mv)	23.4	4.1
Entrapment efficiency (%)	/	>90

b

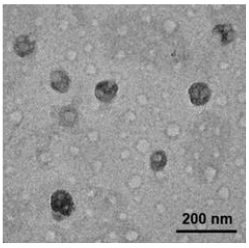

c

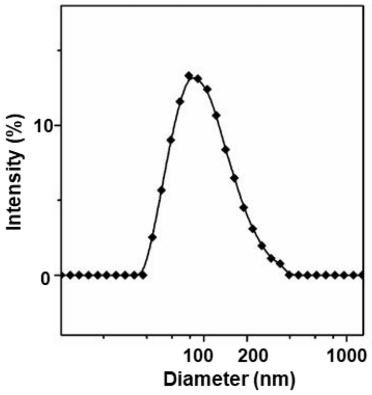

d

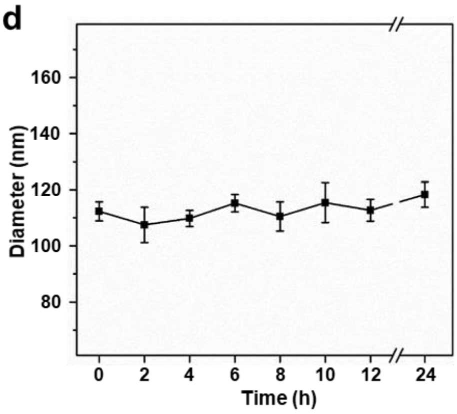

Supplementary Figure 6. The preparation of nanoparticles. a The prepared nanoparticles demonstrated >90% entrapment efficiency for loading siRNA with a Zeta-potential of 4.1 mv. **b** A representative image taken by transmission electron microscope (TEM). **c-d** A normal distribution of hydrodynamic diameter of those nanoparticles with continuous stability within 24 hours (n = 3). The data are represented as the mean ± SEM. Source data are provided as a Source Data file.

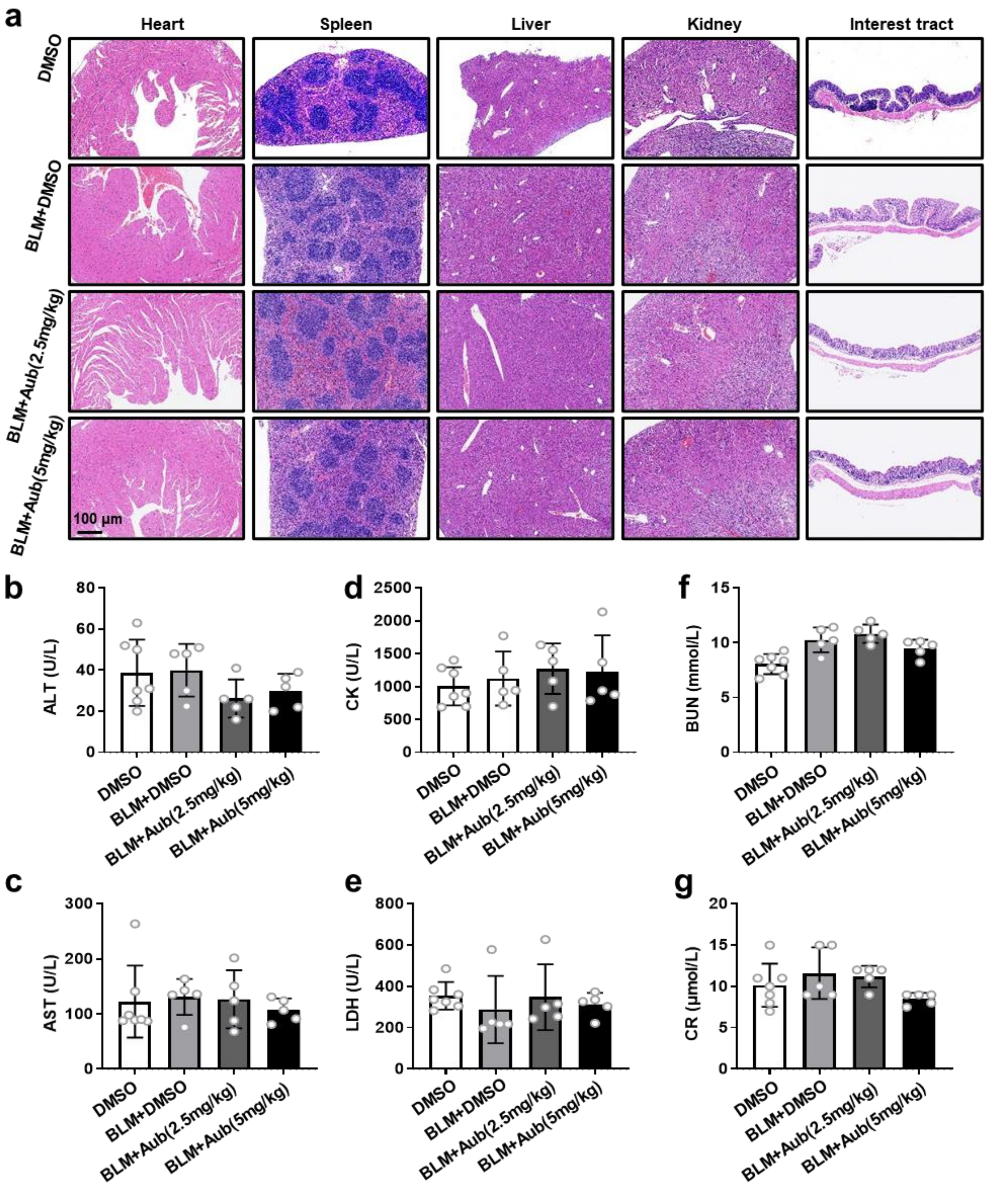

Supplementary Figure 7. The effection of nanoparticles in other organs. a Histological analysis of heart, spleen, liver, kidney, and intestine tract in mice (n = 5) after AubipyOMe injection. Representative images for H&E. Images were captured at ×200 magnification. **b-g** liver (**b-c**), cardiac (**d-e**), and renal (**f-g**), function of mice (n = 5) after AubipyOMe injection. The data are represented as the mean ± SEM. ALT: Alanine aminotransferase; AST: Aspartate aminotransferase; CK: Creatine Kinase. LDH: lactate dehydrogenase; BUN: Blood Urea Nitrogen; CR: Creatinine. Source data are provided as a Source Data file.

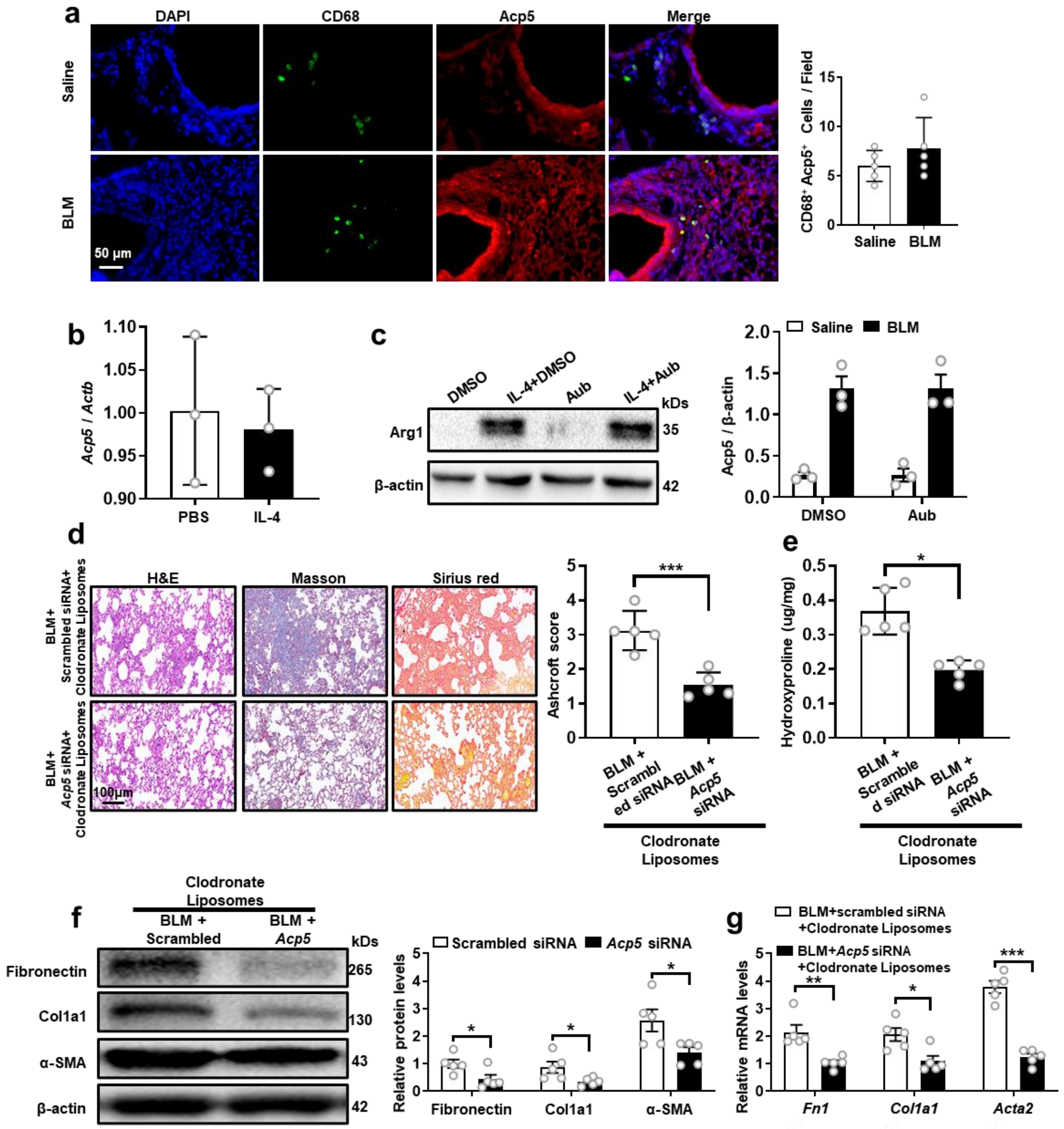

Supplementary Figure 8. The effects of Acp5 on macrophages activation. a Results for co-immunostaining of Acp5 and CD68 in the lung sections from Saline (n = 5) and BLM-induced (n = 5, p = 0.2825) male C57BL/6 mice (8 weeks). The nuclei were stained blue by DAPI, and the images were taken under original magnification ×400. **b** RT-PCR analysis of the levels of *Acp5* in BMDMs following IL-4 (10 ng/ml) treatment (p = 0.7178). **c** Western blot analysis of Arginase 1 in AubipyOMe treated BMDMs following IL-4 stimulation (IL-4+DMSO versus IL-4+Aub: p > 0.9999). **d** Histological analysis of the severity of lung fibrosis in BLM-induced mice after

Clodronate liposome induction. Left panel: representative images for H&E (left), Masson staining (middle) and Sirius red (right). Right panel: A bar graph showed the quantitative mean score of the severity of fibrosis ($p = 0.0008$). **e** Quantification of hydroxyproline contents ($p = 0.0079$). **f-g** Western blot (**f**) and RT-PCR (**g**) analysis of Fibronectin (**f**: $p = 0.0334$, **g**: $p = 0.0079$), Col1a1 (**f**: $p = 0.0429$, **g**: $p = 0.0134$), and α-SMA (**f**: $p = 0.0303$, **g**: $p = 0.0079$) expression. The data are represented as the mean ± SEM and 5 mice were included in each study group. Two-sided Student's *t*-test (a, b, d, f) test and two-tailed Mann–Whitney test (c, e, g) was applied. *, $p < 0.05$; **, $p < 0.01$; ***, $p < 0.001$. Arg1: Arginase 1; BMDMs Bone marrow-derived macrophages; Source data are provided as a Source Data file.

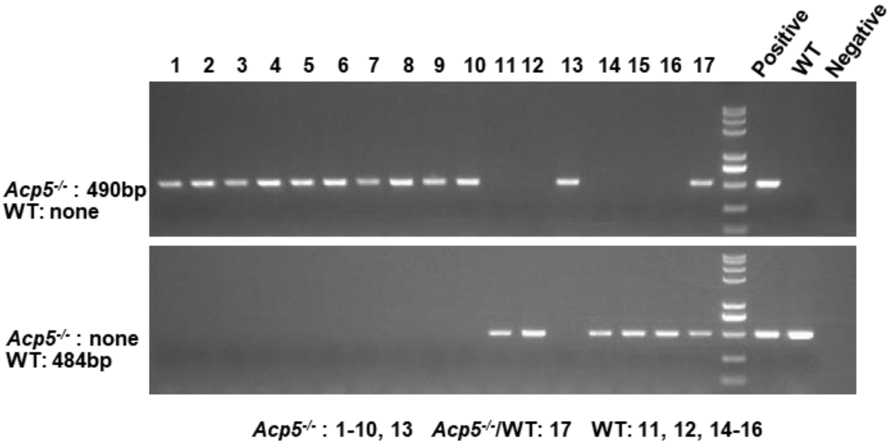

Supplementary Figure 9. Genotyping results of WT and *Acp5*$^{-/-}$ allele. The wildtype allele is none and the *Acp5*$^{-/-}$ allele is 490 bp (up). The wildtype allele is 484 bp and the *Acp5*$^{-/-}$ allele is none (below). The data are represented as the representative image of three independent experiments. Source data are provided as a Source Data file.

Supplementary Table 1. Characteristics of the Patients at Baseline

Variable	Serum samples IPF patients (n = 20)	Serum samples Control subjects (n = 13)	p-Value	Lung tissue samples IPF patients (n = 5)	Lung tissue samples Control subjects (n = 5)	p-Value
Age, years (mean ± SD)	64.30 ± 12.84	59.00 ± 13.74	0.268	56.67 ± 4.096	58.33 ± 3.844	0.7815
Gender						
Male	12 (60%)	6 (46.15%)	0.493	2 (66.7%)	2 (66.7%)	
Female	8 (40%)	7 (53.85%)		1 (33.3%)	1 (33.3%)	
Pulmonary Function						
FVC, % predicted	70.06 ± 13.26	NA		60.33 ± 16.42	NA	
DLCO, % predicted	48.25 ± 18.18	NA		38.25 ± 14.89	NA	

IPF, Idiopathic pulmonary fibrosis; FVC, Forced vital capacity; DLCO, diffusing capacity of the lung for carbon monoxide.

Supplementary Table 2. The primer sequences for RT-PCR

human *FN1*	forward	5′- GAG AAT AAG CTG TAC CAT CGC AA -3′
	reverse	5′- CGA CCA CAT AGG AAG TCC CAG -3′
human *COL1A1*	forward	5′- GAG GGC CAA GAC GAA GAC ATC -3′
	reverse	5′- CAG ATC ACG TCA TCG CAC AAC -3′
human *ACTA2*	forward	5′- GAC GCT GAA GTA TCC GAT AGA ACA CG -3′
	reverse	5′- CAC CAT CTC CAG AGT CCA GCA CAA T -3′
human *ACTB*	forward	5′- AGC GAG CAT CCC CCA AAG TT -3′
	reverse	5′- GGG CAC GAA GGC TCA TCA TT -3′
mouse *Acp5*	forward	5′- CCT GAG ATT TGT GGC TGT GG -3′
	reverse	5′- TCT TGT CGC TGG CAT CGT G -3′
mouse *Fn1*	forward	5′- GAT GTC CGA ACA GCT ATT TAC CA -3′
	reverse	5′- CCT TGC GAC TTC AGC CAC T -3′
mouse *Col1a1*	forward	5′- TAA GGG TCC CCA ATG GTG AGA -3′
	reverse	5′- GGG TCC CTC GAC TCC TAC AT -3′
mouse *Acta2*	forward	5′- GGA CGT ACA ACT GGT ATT GTG C -3′
	reverse	5′- TCG GCA GTA GTC ACG AAG GA -3′
mouse *β-catenin*	forward	5′- TCC CAT CCA CGC AGT TTG AC -3′
	reverse	5′- TCC TCA TCG TTT AGC AGT TTT GT -3′
mouse *Actb*	forward	5′- GCC ACA GCA CTC CAT CGA C -3′
	reverse	5′- GTC TCC GAT CTG GAA AAC GC -3′
