# Supplementary material for: Tartrate-resistant acid phosphatase 5 promotes pulmonary fibrosis by modulating β-catenin signaling
Source: Nat Commun. 2022 Jan 10;13:114. doi: 10.1038/s41467-021-27684-9 (PMC8748833; doi:10.1038/s41467-021-27684-9)
Supplement: Supplementary file 2 — Reporting Summary [file 41467_2021_27684_MOESM2_ESM.pdf]

nature portfolio

Corresponding author(s): Yi Wang and Weining Xiong

Last updated by author(s): Nov 5, 2021

Reporting Summary

Nature Portfolio wishes to improve the reproducibility of the work that we publish. This form provides structure for consistency and transparency in reporting. For further information on Nature Portfolio policies, see our Editorial Policies and the Editorial Policy Checklist.

Statistics

For all statistical analyses, confirm that the following items are present in the figure legend, table legend, main text, or Methods section.

n/a	Confirmed	
☐	☒	The exact sample size (*n*) for each experimental group/condition, given as a discrete number and unit of measurement
☐	☒	A statement on whether measurements were taken from distinct samples or whether the same sample was measured repeatedly
☐	☒	The statistical test(s) used AND whether they are one- or two-sided<br>*Only common tests should be described solely by name; describe more complex techniques in the Methods section.*
☒	☐	A description of all covariates tested
☐	☒	A description of any assumptions or corrections, such as tests of normality and adjustment for multiple comparisons
☐	☒	A full description of the statistical parameters including central tendency (e.g. means) or other basic estimates (e.g. regression coefficient) AND variation (e.g. standard deviation) or associated estimates of uncertainty (e.g. confidence intervals)
☒	☐	For null hypothesis testing, the test statistic (e.g. *F*, *t*, *r*) with confidence intervals, effect sizes, degrees of freedom and *P* value noted<br>*Give P values as exact values whenever suitable.*
☒	☐	For Bayesian analysis, information on the choice of priors and Markov chain Monte Carlo settings
☒	☐	For hierarchical and complex designs, identification of the appropriate level for tests and full reporting of outcomes
☒	☐	Estimates of effect sizes (e.g. Cohen's *d*, Pearson's *r*), indicating how they were calculated

Our web collection on statistics for biologists contains articles on many of the points above.

Software and code

Policy information about availability of computer code

Data collection	SPSS 25.0 (IBM, Armonk, NY, USA), GraphPad Prism 8.0 (San Diego, CA, USA),
Data analysis	All statistical analyses were performed using GraphPad Prism 8.0 (San Diego, CA, USA) or SPSS 25.0 (IBM, Armonk, NY, USA). Correlations between ACP5 expression and the clinical pathological features of IPF patients and control subjects were analyzed by the χ2 test or Fisher's exact test. Other data are expressed as the mean ± SEM, and an independent Student's t-test was administered to analyze the statistical significance of differences between two groups. p<0.05 was used to indicate statistical significance. All data were tested for normalization before analysis.

For manuscripts utilizing custom algorithms or software that are central to the research but not yet described in published literature, software must be made available to editors and reviewers. We strongly encourage code deposition in a community repository (e.g. GitHub). See the Nature Portfolio guidelines for submitting code & software for further information.

Data

Policy information about availability of data

All manuscripts must include a data availability statement. This statement should provide the following information, where applicable:

- Accession codes, unique identifiers, or web links for publicly available datasets
- A description of any restrictions on data availability
- For clinical datasets or third party data, please ensure that the statement adheres to our policy

All data generated in this study are provided in the Supplementary Information/Source Data file. Additional detailed information is available from the corresponding author on reasonable request.
Please consider making the additional information available in a publicly accessible repository, or explain to the editor why this data can only be made available

from the corresponding author on reasonable request.

Field-specific reporting

Please select the one below that is the best fit for your research. If you are not sure, read the appropriate sections before making your selection.

☒ Life sciences ☐ Behavioural & social sciences ☐ Ecological, evolutionary & environmental sciences

For a reference copy of the document with all sections, see nature.com/documents/nr-reporting-summary-flat.pdf

Life sciences study design

All studies must disclose on these points even when the disclosure is negative.

Sample size	No statistical method was used to pre-select the sample size. The sample size was determined based on similar previous studies of our labratory and on previous experiments using similar methodologies. Detailed sample size were described in the figure legends.
Data exclusions	We have no data were excluded from the analyses.
Replication	Experimental findings were reliably reproduced in at least three independent experiments.
Randomization	Mice were stratified according to body weight and then randomized into the different groups. Cells and tissues from human donors were also randomized.
Blinding	The investigators were blinded to group allocation during data collection and analysis.

Reporting for specific materials, systems and methods

We require information from authors about some types of materials, experimental systems and methods used in many studies. Here, indicate whether each material, system or method listed is relevant to your study. If you are not sure if a list item applies to your research, read the appropriate section before selecting a response.

Materials & experimental systems

n/a	Involved in the study	
☐	☒	Antibodies
☒	☐	Eukaryotic cell lines
☒	☐	Palaeontology and archaeology
☐	☒	Animals and other organisms
☐	☒	Human research participants
☒	☐	Clinical data
☒	☐	Dual use research of concern

Methods

n/a	Involved in the study	
☒	☐	ChIP-seq
☒	☐	Flow cytometry
☒	☐	MRI-based neuroimaging

Antibodies

Antibodies used

anti-ACP5 (Gentex GTX100438, CA, USA, 1:1,000);
mouse anti-ACP5 (Abnova H00000054-M01, Taipei, China, 1: 100),
rabbit anti-ACP5 (Proteintech 11594-1-AP, Wuhan, China, 1:100),
rabbit anti-FSP1 (Proteintech 16105-1-AP Wuhan, China, 1:100),
anti-LAMIN B1(Proteintech 12987-1-AP, Wuhan, China, 1:1,000),
anti-FIBRONECTIN (Proteintech 15613-1-AP, Wuhan, China, 1:1,000),
anti-COL1A1 (Proteintech 67288-1-lg, Wuhan, China, 1:1,000),
anti-α-SMA(Cell Signaling Technology 19245, MA, USA, 1: 1000),
anti-phospho-β-CATENIN (Ser33/37/Thr41) (Cell Signaling Technology 9561, MA, USA, 1: 1000),
anti-β-CATENIN (Cell Signaling Technology 8480, MA, USA, 1: 1000);
anti-Pcna (Cell Signaling Technology 13110, MA, USA, 1:100),
mouse anti-α-SMA (Cell Signaling Technology 48938, MA, USA, 1:100),
rabbit anti-phospho-smad3 (Cell Signaling Technology 9520, MA, USA, 1:100).
anti-GAPDH(Abcam ab8245, MA, USA, 1:3000).
anti-β-ACTIN (Abcam ab8226, MA, USA, 1:3000).
UltraPolymer Goat anti-Mouse IgG（H&L）-HRP (Proteintech PR30012, Wuhan, China, 1:1000).
UltraPolymer Goat anti-Rabbit IgG（H&L）-HRP (Proteintech PR30011, Wuhan, China, 1:1000).
Alexa 488-conjugated anti-mouse (A23210 Abbkine, CA, USA, 1:400);
Alexa 594-conjugated anti-rabbit (A23420 Abbkine, CA, USA, 1:400);

	Alexa 488-conjugated anti-mouse (A23210 Abbkine, CA, USA, 1:400);<br>Alexa 594-conjugated anti-rabbit (A23420 Abbkine, CA, USA, 1:400);
Validation	All antibodies that are commercially available were used according to manufactures instructions. Every antibody was validated in the lab to exclude non-specific signals due to secondary antibodies using as positive control.<br>anti-ACP5, https://www.antibodypedia.com/gene/25921/ACP5/antibody/169889/GTX100438;<br>mouse anti-ACP5, http://www.abnova.com/products/products_detail.asp?catalog_id=H00000054-M01;<br>rabbit anti-ACP5, http://www.ptgcn.com/products/ACP5-Antibody-11594-1-AP.htm;<br>rabbit anti-FSP1, http://www.ptgcn.com/products/S100A4-Antibody-16105-1-AP.htm;<br>anti-LAMIN B1, http://www.ptgcn.com/products/LMNB1-Antibody-12987-1-AP.htm;<br>anti-FIBRONECTIN, http://www.ptgcn.com/products/FN1-Antibody-15613-1-AP.htm;<br>anti-COL1A1, http://www.ptgcn.com/products/Collagen-I-Antibody-67288-1-Ig.htm;<br>anti-α-SMA, https://www.cellsignal.cn/products/primary-antibodies/a-smooth-muscle-actin-d4k9n-xp-rabbit-mab/19245?site-search-type=Products&N=4294956287&Ntt=19245&fromPage=plp&_requestid=1610353;<br>anti-phospho-β-CATENIN (Ser33/37/Thr41), https://www.cellsignal.cn/products/primary-antibodies/phospho-b-catenin-ser33-37-thr41-antibody/9561?site-search-type=Products&N=4294956287&Ntt=9561&fromPage=plp&_requestid=1610421;<br>anti-β-CATENIN, https://www.cellsignal.cn/products/primary-antibodies/b-catenin-d10a8-xp-rabbit-mab/8480?site-search-type=Products&N=4294956287&Ntt=8480&fromPage=plp&_requestid=1610481;<br>anti-Pcna, https://www.cellsignal.cn/products/primary-antibodies/pcna-d3h8p-xp-rabbit-mab/13110?site-search-type=Products&N=4294956287&Ntt=13110&fromPage=plp&_requestid=1610543;<br>mouse anti-α-SMA, https://www.cellsignal.cn/products/primary-antibodies/a-smooth-muscle-actin-1a4-mouse-mab-if-formulated/48938?site-search-type=Products&N=4294956287&Ntt=48938&fromPage=plp&_requestid=1610600;<br>rabbit anti-phospho-smad3, https://www.cellsignal.cn/products/primary-antibodies/phospho-smad3-ser423-425-c25a9-rabbit-mab/9520?site-search-type=Products&N=4294956287&Ntt=9520&fromPage=plp&_requestid=1603793;<br>anti-GAPDH, https://www.abcam.cn/gapdh-antibody-6c5-loading-control-ab8245.html;<br>anti-β-ACTIN, https://www.abcam.cn/beta-actin-antibody-mabcam-8226-loading-control-ab8226.html.

Animals and other organisms

Policy information about studies involving animals; ARRIVE guidelines recommended for reporting animal research

Laboratory animals	Male mice (C57BL/6 background, 8-10 weeks) were used in this study. Housing conditions:Temperature:20-24°C; Humidity:45-65%, 12/12 hours light/dark cycle.
Wild animals	This study did not involve wild animals
Field-collected samples	This study did not involve field-collected animals
Ethics oversight	All experimental procedures were approved by the Animal Care and Use Committee at Tongji Hospital.

Note that full information on the approval of the study protocol must also be provided in the manuscript.

Human research participants

Policy information about studies involving human research participants

Population characteristics	We have described the information in the supplementary table 1.
Recruitment	Participants in the control subjects came from relatively healthy patients who voluntarily came to hospital for physical examination. The sera and tissues from subjects were collected at Tongji Hospital. An IPF diagnosis was made according to consensus diagnostic criteria from the European Respiratory Society (ERS)/American Thoracic Society (ATS). Written informed consents were obtained from all subjects. There is no self-selection bias involved.
Ethics oversight	Study protocol was approved by the Ethics Committee according to guidelines of the 1975 Declaration of Helsinki. The experiments were approved by the Human Assurance Committee of Tongji Hospital.

Note that full information on the approval of the study protocol must also be provided in the manuscript.
